# Ratio-Driven Lipoprotein Mapping Refines Genetic Pathways of Cardiometabolic Risk

**DOI:** 10.21203/rs.3.rs-8475327/v1

**Published:** 2026-01-07

**Authors:** Karsten Suhre, Murugan Subramanian, Melanie Modder, Abulaish Anasari, Haiyue He, Christopher Krumm, Farooq Rashid, Raghad Al-Ishaq, Aziz Belkadi, Tanwir Habib, Anna Halama, Nisha Stephan, Gaurav Thareja, Shaza Zaghlool, Audrey D Dujardin, Xi Chen, Peter Mulligan, Eric B. Fauman, Ann-Hwee Lee, Patrick C. N. Rensen, Sander Kooijman, David E Cohen, S. Hani Najafi-Shoushtari

**Affiliations:** 1 Bioinformatics Core, Weill Cornell Medicine-Qatar, Education City, 24144 Doha, Qatar.; 2 Department of Biophysics and Physiology, Weill Cornell Medicine, New York, NY, USA.; 3 Department of Cell and Developmental Biology, Weill Cornell Medicine, New York, NY, USA.; 4 Division of Research, Weill Cornell Medicine-Qatar, Education City, 24144 Doha, Qatar.; 5 Division of Gastroenterology and Hepatology, Weill Cornell Medicine, New York, NY, USA.; 6 Department of Pathology and Laboratory Medicine, Weill Cornell Medicine, New York, NY 10065, USA.; 7 Department of Medicine, Division of Endocrinology, Leiden University Medical Center, Leiden, The Netherlands.; 8 Centre Léon Bérard, Cancer Research Center of Lyon, Université de Lyon, Université Claude Bernard Lyon 1, INSERM 1052, CNRS 5286, Lyon, France.; 9 Department of Molecular and Cellular Biology, Lester and Sue Smith Breast Center, and Dan L. Duncan Cancer Center, Baylor College of Medicine, Houston, Texas, USA.; 10 Internal Medicine Research Unit, Pfizer Worldwide Research, Development and Medical, Cambridge, MA, 02139, USA.

**Keywords:** lipid and cholesterol metabolism, lipoprotein particle composition, cardiovascular disease, genetic association, drug target development, lipo-proteomics, microRNA

## Abstract

Dysregulated blood lipids are a major predictor of cardiovascular events. A recent genome-wide association study (GWAS) with five clinically relevant lipid traits in 1.65 million individuals implicated over 770 genomic regions in regulating blood lipid metabolism. To translate these associations into clinical applications, a functional understanding of their roles in lipoprotein metabolism, transport and remodeling (LPmtr) is required. Here, we report the deep molecular fine-mapping of 554 of these lipid risk loci using 168 lipoprotein-related traits and all possible ratios between them in over 273,000 participants of the UK Biobank. We identified new ratio-based markers of pathways shared by multiple LPmtr genes, such as the linoleic acid fraction of the polyunsaturated fatty acid pool to reveal potential causal genes at poorly characterized lipid risk loci, the percentage of esterified cholesterol moieties in LDL particles as a proxy for soluble LDL receptor levels, and the HDL fraction of total lipoprotein particle number as a predictor of incident myocardial infarction. We demonstrate how lipoprotein fine-mapping can generate new hypotheses for drug target development while uncovering new mechanisms relevant to hyperlipidemia. Ratio-driven clustering further implicated miR-148 in TG secretion, linking ER-stress responses at postprandial state to VLDL metabolism via mTORC1, shown through series of integrated cellular assays and mouse studies. Moreover, consistent with its regulatory influence on lipid flux we identify miR-148a a previously unrecognized determinat of **Lp(a) levels**. Our study implements a novel approach of using metabolomic data to follow-up on genetic evidence from GWAS with clinical traits and generates new insights into the biology of lipoprotein particles, supporting the emerging view that assessing lipoprotein size and composition is essential for the understanding, prevention, and treatment of lipid-related disorders.

## INTRODUCTION

Improper trafficking of plasma cholesterol, triglycerides (TG) and phospholipids in lipoprotein particles lies at the heart of metabolic dysregulation and is associated with major cardio-metabolic disease indications, including atherosclerotic cardiovascular disease (ASCVD) and non-alcoholic fatty liver diseases^1^. Over the last decade, genome-wide association studies (GWAS) identified many common genetic variants that associate with major clinical lipid traits^2–4^, suggesting potential therapeutic targets^5^, supporting drug target prioritization^6^, and disentangling causal relationships by Mendelian randomization^7,8^. Recently, Graham *et al*.^9^ published a GWAS with lipid levels in a multi-ethnic study with 1.65 million individuals, including 350,000 participants of non-European ancestries. In what is arguably the largest GWAS on lipid traits to date, they reported over 770 genomic regions that were associated with the five “classical” blood lipid traits currently used in clinical practice, that is, LDL-cholesterol (LDL-C), HDL-cholesterol (HDL-C), TG, total cholesterol (TC), and non-HDL-C^9^.

While the genetic architecture of these lipid-risk loci has been mapped out in detail in the Graham *et al*.^9^ study, much less is known about their metabolic pleiotropy, their role in lipoprotein metabolism, and eventually their potential contribution to ASCVD related pathologies. Genetic associations with detailed lipoprotein properties, such as particle size-resolved core lipid content, can provide a deeper understanding of how these lipid-risk genes are involved in the processes that control lipoprotein metabolism, transport, and remodeling (LPmtr)^10^. However, only a few GWAS with size- and composition-resolved lipoprotein traits truly account for the intricate nature of these outcomes in their biochemical interpretation of the identified genetic associations.

Here we leverage the recently published Nightingale NMR data that is presently available for ~280,00 participants of the UK Biobank (UKB) to fine-map the metabolic architecture of these genetic lipid risk loci using a comprehensive set of 168 lipoprotein related metabolic traits^11–14^. The Nightingale nuclear magnetic resonance (NMR) platform covers the lipid composition of fourteen VLDL, IDL, LDL, and HDL size classes, quantifying their respective free and esterified cholesterol, TG, and phospholipid content (Tables S1 & S2). Motivated by past successes of analyzing ratios with metabolomic and proteomics traits^15–18^ we hypothesize that ratios between lipoprotein traits may reveal proxies of shared biochemical pathways and non-genetic variances. In contrast to the hypothesis-free GWAS approach taken by other studies^19–22^ we focus on the already established lipid risk genes reported by Graham *et al*.^9^ (Table S3).

To validate the platform, we start by replicating these lipid risk genes using the same five lipid traits as used in the original study but measured on the Nightingale platform in 231,145 samples that are classified as genetically “Caucasian” [sic] by UKB. We then proceed to their molecular fine mapping using the 168 NMR traits as endpoints using twice as many samples as the largest previously published study^22^. Finally, we conduct a hypothesis-free all-against-all ratio-metric association study with 14,196 ratios between all lipoprotein traits for all genetic lipid risk variants reported by Graham *et al*.^9^. Throughout, we replicate our findings using data from the ethnically diverse set of 43,214 genetically non-”Caucasian” participants in UK Biobank.

We show that increasing the phenotype space from the five “classical” lipids to 168 NMR traits and then to 14,196 ratios not only provides relevant new insights into the biological function of the associated lipid risk loci, but also broadly sharpens the association signals by increasing the statistical power to detect significant genetic associations with these phenotypes. We identify specific ratios between NMR traits as proxies for biochemical processes shared by multiple genes and investigate their biomedical relevance. Finally, we demonstrate that our approach can lead to the identification and characterization of novel therapeutic targets by following-up with experimental evidence for a novel microRNA that modulates VLDL traits (Figure S1).

## RESULTS

### A total of 274 lipid risk loci from a seven-times larger GWAS were confirmed using the Nightingale platform.

Graham *et al*.^9^ reported 2,624 genetic associations on 1,835 lead variants with five major lipid traits (TC, TG, LDL-C, HDL-C, non-HDL-C) at over 770 genomic regions (Supplementary Table 3 in Graham *et al*.). Their associations include conditional analyses with up to 46 variants in the model and were conducted in multiple ancestries. We obtained genotype data for all but five of these variants from the UKB research analysis platform (RAP) and computed their associations with 168 NMR-derived traits and also all 14,196 ratios between these traits in linear models including the lead variants, age, age^2^, sex, use of lipid lowering medication, the ten first genotype principal components, and all respective conditional genetic variants reported by Graham *et al*. as covariates.

While the 2,624 conditioned models used by Graham *et al*. are sometimes different for the five lipid traits, their lead variants could be identical or in high LD, effectively representing a same genetic signal. We therefore limited our analysis to lead variants that were not in high LD (r^2^ < 0.7), and in cases where multiple traits and conditional variants were reported for a same lead variant, we kept the model with the strongest association signal to the five lipid traits in Graham *et al.*. To avoid spurious associations with rare variants, we further limited our analysis to variants with minor allele frequencies (MAF) of >1% and to associations that were genome-wide significant (p<10^−8^) in the conditioned analysis by Graham *et al*. A total of 1,054 variants satisfied these criteria and constituted the starting point for the following analyses (Table S3).

Although we conducted association tests on a targeted set of 1,054 gene variants, we chose to apply a genome-wide significance threshold of p_Bonf_ = 5×10^−8^ so that our conclusions hold if they were conducted in a GWAS context. We further required conservative Bonferroni significance throughout by also accounting for the number of traits tested as appropriate, that is, by dividing p_Bonf_ by 5 (p_ref_) for testing five lipids, by 168 (p_NMR_) for testing all NMR traits, and by 14,196 (p_AllRatios_) for testing all possible NMR ratios. To avoid rounding small p-values to zero, we report negative log10-scaled p-values throughout.

A total of 274 (26.0%) out of the 1,054 primary associations from Graham *et al*. were significant at p_ref_ for at least one of the five lipid traits and can therefore be considered replicated in UKB using the Nightingale platform, and this despite the fact that our cohort size was only 15% that of the discovery study. Note that we observed no substantial difference when using lipid data from clinical biochemistry (Figure S2), indicating that the two data sets are of comparable quality and that the replication rate is largely determined by sample size and potentially differences in the study population, but not by differences in the readouts of the clinical biochemistry and the Nightingale platforms.

### Metabolic fine mapping using 168 NMR traits increased the number of identified lipid risk loci by 62.8%.

We then tested all of the Graham et al. lipid risk variants for association with the 168 NMR traits and found that 446 (42.3%) of the 1,054 lead variants were associated at a Bonferroni significance level with at least one of the 168 Nightingale traits. In other words, of the 780 variants that did not replicate with any of the five “classical” lipid traits, 179 were significantly associated (p < p_NMR_) with at least one of the other NMR traits. Hence, despite the increased multiple-testing burden by using the NMR traits as endpoints, their inclusion led to the discovery of 62.8% additional variants (446 versus 274), indicating that these detailed lipoprotein traits carry information that is not captured by the five “classical” lipid traits. We report the strongest association with each variant in Table S3 and share the complete summary statistics for all genetic associations with all traits as Supplementary Data.

**Figure 1:**
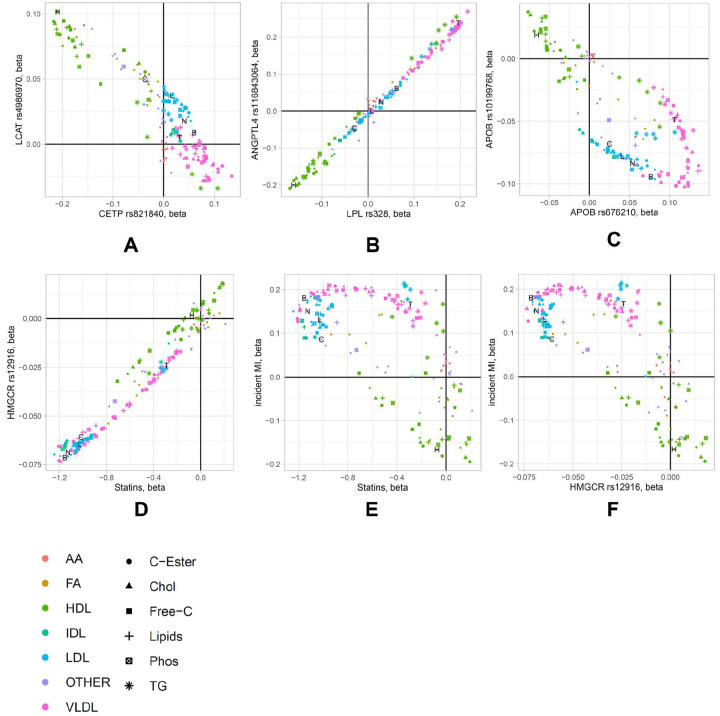
Application of NMR association profiles for drug target assessment. Scatterplot of the effect estimates for the association of variant rs4986970 near LCAT with the 168 NMR traits against the associations of variant rs4986970 near LCAT (A); same for rs328 near LPL against rs11843064 near ANGPTL4 (B); two uncorrelated variants (rs676210 and rs10199768) at the APOB locus (C), rs12916 near HMGCR against the effect of statin usage, based on 35,601 participants reporting statin usage (D); statin usage against incident myocardial infarction (E); and rs12916 near HMGCR against myocardial infarction; NMR traits are colored by type, the lipid content of the respective lipoprotein particles is indicated by the symbols; Blood levels of **H**DL-C, **L**DL-C, **C**holesterol, **T**riglycerides, and Apolipoprotein **B** are indicated by the letters H, L, C, T, and B, respectively.

Hypothesizing that gene loci with similar NMR association profiles share common biology, we visualized the association strengths (-log_10_(p-value)) and effect sizes (aka betas) as clustered heatmaps (Figure S1B and Tables S4 & S5) and confirmed that many well studied genes that act on a same pathway clustered closely together and induce similar responses in their lipoprotein composition to genetically induced perturbations, such as LCAT (lecithin:cholesterol acyltransferase) and CETP (cholesteryl ester transfer protein), which are both plasma proteins critical to HDL maturation and remodeling^23^ (Figure 1A), or ANGPTL4 (angiopoietin-like 4), a secretory protein that restricts LPL (lipoprotein lipase) activity to limit plasma triglyceride-rich lipoprotein-TG hydrolysis and subsequent release of fatty acids for uptake into adipose tissue during fasting, which had a profile similar to LPL^24^ (Figure 1B).

The agreement between the effect of genetic variance in HMGCR and the effect of statin treatment on the NMR traits (Figure 1D–F) is a textbook example of how genetic associations can provide a proxy for the effect of therapeutically modifying the corresponding protein’s level or activity^11,25^. Here we report three additional examples of genetic associations of the genes coding for the targets of beta blockers, ezetimibe, and glitazones (ADRB1, NPC1L1, PPARG, resp.) with NMR traits (Figure S3). However, the relationship between the effects of these genetic variants and of the corresponding drugs on the NMR traits are more complex than in the case of statins, suggesting that these variants are not perfect proxies for the related therapeutic intervention. At the example of APOB, we further report a case where two variants at the same gene locus yield diametrically different association patterns with the NMR traits. One variant (rs676210) is protein altering while the other (rs138905573) is located upstream of APOB; we speculate that the former may change the physical properties of APOB binding to the LDL receptor while the latter may possibly regulate APOB protein levels, leading to different consequences on the NMR profiles. Taken together, these examples outline how lipoprotein fine-mapping of the Graham *et al*. GWAS lipid risk loci can now be used for hypothesis generation in the framework of drug target discovery and development, but they also highlights the complexity of using genetic variation as predictors of the potential outcome of therapeutic intervention.

### Hypothesis-free testing of all ratios between NMR traits further increased the number of significant lipid risk loci by 25.6%.

We then computed the associations of all 14,196 possible ratios between the 168 NMR traits with all lipid risk variants and identified 554 significant associations (52.6%) with ratios (p-value < p_AllRatios_). Of the 608 Graham *et al*. variants that were not associated with at least one of the NMR traits, 114 became significant with at least one ratio. On the other hand, only six variants that were significant with NMR traits did not reach the higher significance level required when using ratios. Hence, testing all possible ratios rather than only the NMR traits allowed us to identify 114 additional loci, while losing only six loci with weaker signals due to the increased multiple testing burden (Figure S1C).

We used the p-gain statistics to identify ratios that increased the strength of an association above the signal already carried by the two individual traits^17,18^. The p-gain is defined as the smaller of the two p-values for the trait association divided by the p-value for the association with the ratio. A p-gain of ten is considered significant at an alpha level of 0.05 for a single test. Out of the 446 variants that were associated with an NMR-trait, 327 variants also associated with a ratio with a Bonferroni-significant p-gain (p-gain > p-gain_AllRatios_ = 10 * 10^6^ * 14,196 = 10^11.2^). Hence, the testing of all possible ratios provided new information by linking two NMR traits at already known loci in 73.3% (327/446) of the cases, while at the same time increasing the power to discover new loci by 25.6% (114/446).

### Over 80% of all sufficiently powered genetic associations were replicated in an ethnically diverse cohort.

For replication we used samples from up to 43,214 ethnically diverse participants of the UKB. These samples were labeled as genetically “non-Caucasian” [*sic*] by UKB and were not used in the discovery. We attempted the replication of the lead associations of all NMR trait and ratio associations that reached Bonferroni significance in the discovery study, using a replication significance level of p < 0.05 divided by the number of discovered loci, that is, p < 0.05/446 for traits and p < 0.05/554 for ratios. We computed statistical power to replicate by randomly sampling 43,214 records from the discovery cohort one hundred times and counting how often associations on these random subsets reached the significance level for replication. For associations with NMR traits, out of 163 associations that had >80% replication power we replicated 140 (85.9%) and for associations with NMR ratios, out of 228 variants with >80% replication power we replicated 185 (81.1%), suggesting that essentially all investigated associations are robust, even when analyzed in an ethnically diverse cohort, and that most loci are likely to replicate given sufficient statistical replication power. A reason for this high replication rate resides arguably in the fact that all loci that we investigated here have previously been identified in association with the five classical lipid traits in the much larger study by Graham *et al*., a fact that supports our approach of focusing our phenotypic fine mapping on already known lipid risk loci from a more highly powered GWAS.

### The linoleic acid fraction of the polyunsaturated fatty acid pool indicates potential causal genes at poorly characterized lipid risk loci.

We previously showed that large p-gains are observed when the ratio of two traits represents a sharper readout of the process that is modified by the associated genetic variant than the individual NMR traits on their own^15–18^. We showed that an increase in the strength of an association with ratios can be observed in two situations: (1) when the enumerator and the denominator traits are both controlled by a same variant but in opposite directions, which leads to an increase in the effect size of the association with the ratio, or (2) when one of the two traits in the ratio acts as a proxy for some shared non-genetic confounder that reduces the non-explained variance of the other trait in the association statistics^18^. Note that in the latter situation we do not assume that the normalizing trait is under the influence of the genetic variant. Also, note that spurious associations that might arise due to collider bias are eliminated here by imposing a statistically significant p-gain threshold.

**Figure 2:**
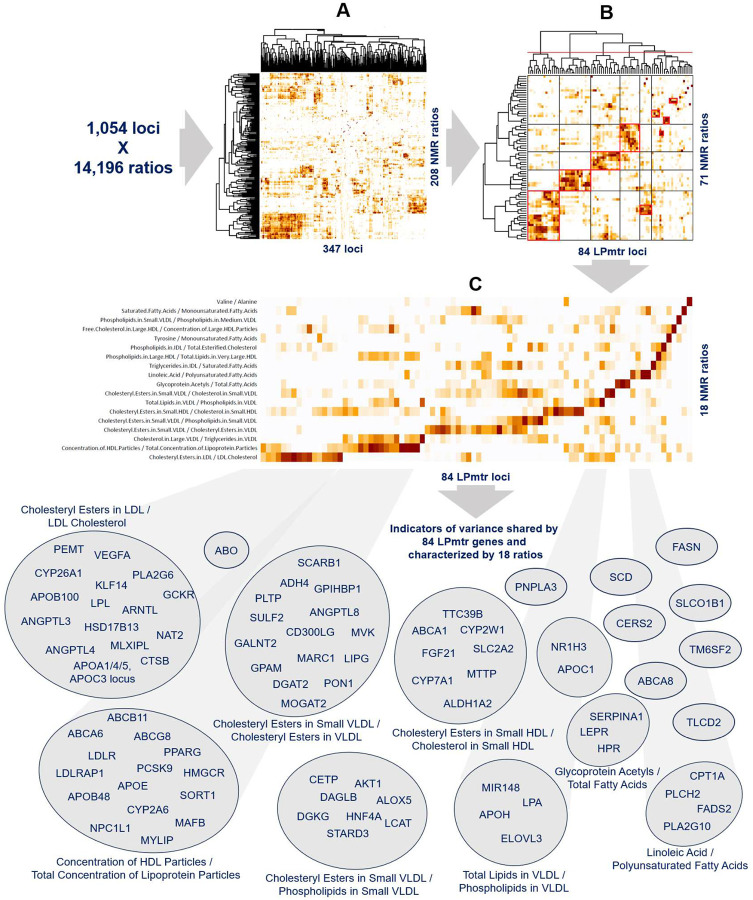
Identification of gene clusters and their corresponding representative ratios. Out of 1,054 lipid risk loci that were tested for association with 14,196 ratios, a total of 347 loci associated at a Bonferroni significant p-gain (p-gain > 10^11.2^) with a ratio, covering 208 ratios that corresponded to a lead association for at least one of these loci; The resulting matrix containing the 208 ratios by 347 gene loci log_10_(p-gain) values of was normalized by the largest log10pgain at each locus and then clustered (**panel A**, data provided in Table S7A); The set of loci was then limited to 84 well annotated gene loci that are all established to be functionally involved in lipoprotein metabolism, transport, and remodeling, together with 71 ratios that associate at one of these loci (**panel B**, data provided in Table S8); Finally, 18 clusters were curated, selecting the most representative ratio for each of them (**panel C**, data provided in Table S9).

We hypothesize that multiple variants that associate with a significant p-gain with a similar set of ratios are impacting genes that act on a shared pathway or are otherwise linked by some shared biochemical process or confounder. Since the NMR traits and also their ratios are highly correlated, we focused on a set of representative ratios by retaining the 208 ratios that corresponded to the lead ratio associations at one of the 347 genetic loci with a Bonferroni significant p-gain (log_10_(p-gain) > 11.2). To account for differences in effect sizes, we normalized the resulting log10pgain matrix at every locus by the largest log10pgain at that locus and then clustered the 208 ratios by 347 loci matrix of normalized p-gains (Figure 2 and Table S7, association data is in Table S8). Clusters of biochemically similar ratios and functionally related genes emerged. We discuss these in the following.

**Figure 3:**
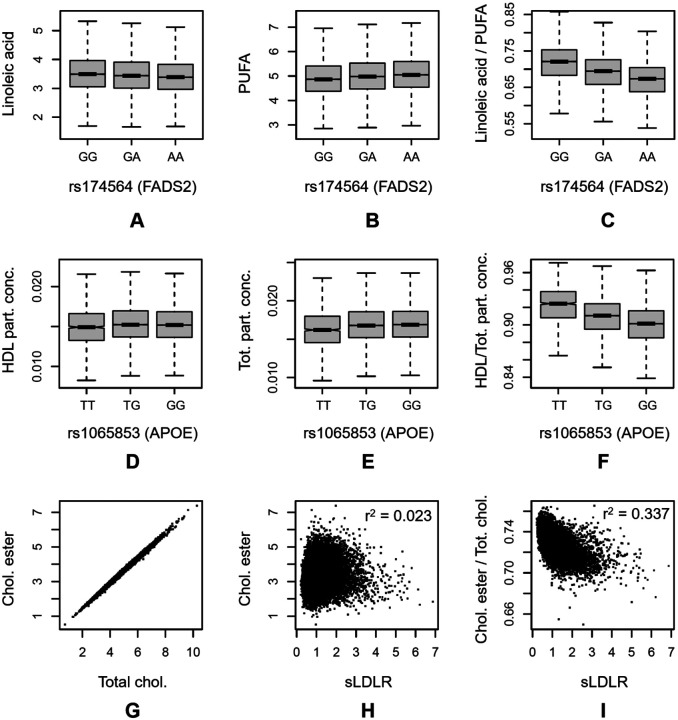
Selected box- and scatter plots. Genetic association of variant rs174564 at the FADS2 gene locus with LA, PUFA, and the LA/PUFA ratio (A-C); Genetic association of variant rs1065853 at the APOE gene locus with the HDL particle concentration, the total lipoprotein particle concentration, and the ratio of both (D-F); Scatterplot of cholesteryl ester and total cholesterol (G), sLDLR and cholesteryl ester (H), and sLDLR and the ratio of cholesteryl ester and total cholesterol (I); Units are mmol/l for all traits except for sLDLR, which is in arbitrary units.

The ratio association with the highest p-gain was rs174564 near *FADS2* (Fatty Acid Desaturase 2) with the ratio of Linoleic Acid (LA) divided by Polyunsaturated Fatty Acids (PUFA), that is, the LA fraction in the PUFA pool (Figure 3A–C). LA is a substrate of the FADS2 enzyme which converts it into γ-linolenic acid, which is then further converted by other enzymes into related PUFAs. The LA / PUFA ratio can therefore be interpreted as a generalized substrate-product pair of the FADS2-catalyzed reaction. In this case, both traits are associated with the rs174564 variant with opposite directionality (log10p__LA_ = 169.6 and log10p__PUFA_ = 282.5). Notably, the association with the ratio is over 4,800 orders of magnitude stronger than the associations with the individual traits (log10p__LA/PUFA_ = 5101.7), reflecting our previous findings from GWAS with metabolomic trait ratios where we identified substrate-product pairs of enzymatic reactions through large p-gains in a hypothesis-free approach^26,27^.

The second strongest association with the LA / PUFA ratio was with rs12928099 and clustered with the *FADS2* association. This locus was annotated by Graham *et al*. with *RRN3, PDXDC1, NTAN1* as candidate genes, but none of these genes are functionally related to PUFA metabolism. We therefore searched the wider genomic region for potentially causal genes and identified *PLA2G10* at a distance of 363kb as a likely candidate. PLA2G10 is a phospholipase that preferentially releases sn-2 bound PUFAs over saturated fatty acids^28^, which is in line with the hypothesis that genetic variation in PLA2G10 activity or expression leads to a shift in the LA / PUFA ratio. This functional assignment is further corroborated by association data from an orthogonal platform, that is, variant rs12928099 was associated in the UK Biobank Olink proteomics GWAS with circulating PLA2G10 protein levels (−log10(p) = 17.3)^29^.

To further validate the hypothesis that the LA / PUFA ratio represents a biochemically relevant marker that allows the identification of causal genes in GWAS, we analyzed all 13 loci that were associated with the LA / PUFA ratio at a significant p-gain (log_10_(p-gain) > 11.2). For all but two of these associations we could identify a clear candidate gene involved in LA and PUFA metabolism (Table 1). In two cases (*PLCH2* and *PLA2G10*) the nearby genes annotated by both, Graham *et al*. and Open Targets^30^, did not include the causal genes and it was the nature of the LA / PUFA ratio that allowed the identification of a putative causal gene. We argue that many of the other ratio associations that we report here (Table S8) can now be used to make similar arguments for the identification of causal genes at less well understood Graham *et al*. lipid risk loci, which is important, as knowledge of the causal genes is a prerequisite for their inclusion into the drug target development pipelines.

### The percentage of esterified cholesterol moieties in individual lipoprotein size classes is a proxy for circulating LDL receptor levels.

To gain further insights into the biological processes underlying the associated ratios, we limited the genetic associations to 84 loci with a clearly identifiable underlying causal gene and an established clear function in lipoprotein metabolism, transport, and remodeling (LPmtr genes). These loci involved 71 lead ratios. We clustered the resulting 71 by 84 matrix (Figure 2B) and then identified 18 clusters, annotating them with the ratio that was most consistently associated with all genes in the respective cluster (Figure 2C).

**Figure 4:**
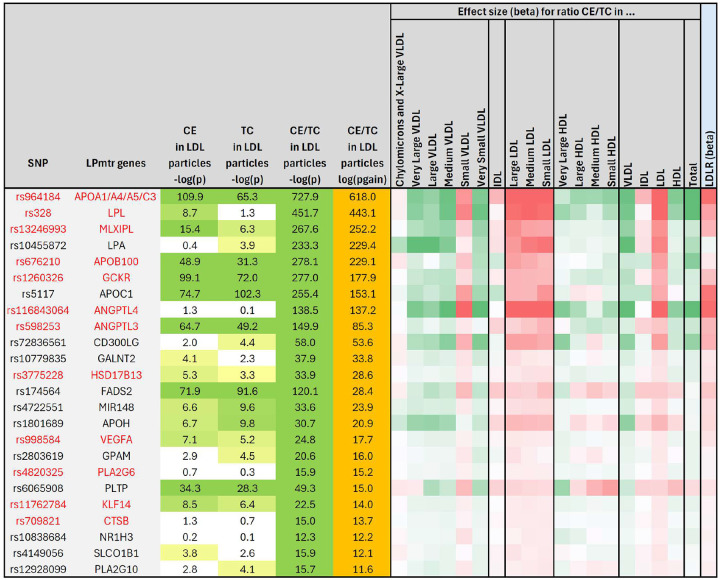
Heatmap of the effect sizes of the genetic associations with CE/TC ratio in the different lipoprotein size classes. P-values and p-gains for all significant associations (p-gain > 10^11.2^) of all LPmtr gene loci with the ratio of cholesteryl ester (CE) in LDL particles divided by total cholesterol (TC) in LDL particles (left); Effect sizes for the associations of the CE/TC ratio in the 14 lipoprotein size classes, grouped by lipoprotein type (VLDL, IDL, LDL, HDL), and total (right); The effect directionality is represented relative to the directionality of the CE/TC ratio in LDL particles; The effect size of the association of the circulating LDLR protein levels with these gene variants are from the UKBPPP Olink study^29^ (plot data is in Table S10); Genes that are part of the CE/TC ratio cluster are in red.

The largest cluster was with genetic variants in 24 LPmtr genes that were associated with a significant p-gain (p-gain > 10^11.2^) with the ratio of cholesteryl ester in LDL particles divided by total cholesterol in LDL particles (CE/TC ratio), that is, the percentage of cholesterol moieties in LDL particles that are esterified with a fatty acyl chain. We then asked whether this association had similar directionality across the lipoprotein size spectrum and found that most of the variants had identical effect directionality within the LDL size classes but opposite effects within the HDL and most of the VLDL size classes, with the notable exception of small VLDL particles, which followed the directionality of the LDL particles (Figure 4). We therefore speculate that the majority of the small VLDL particles may be of a different origin than the other VLDL particles, since they behave more like LDL particles.

**Figure 5:**
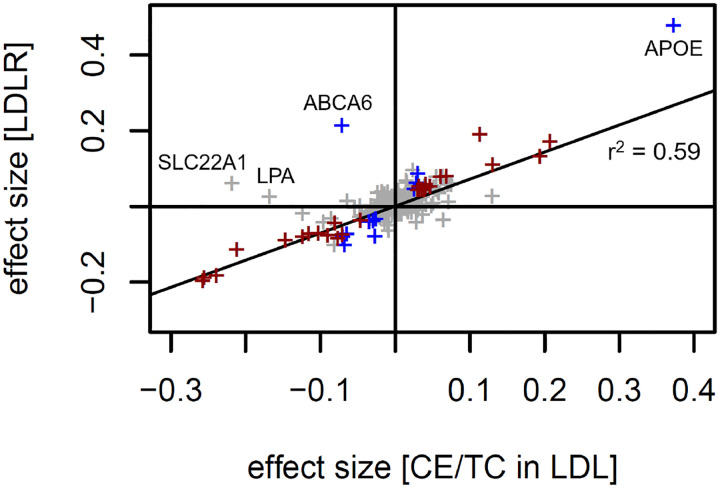
Scatterplot of the effect sizes of the genetic associations with CE/TC in LDL particles and with LDLR levels in the UKBPPP Olink study. Included are the 347 variants that have at least one ratio association with a significant p-gain; Associations that are significant for the association with LDLR (p-value < 5×10^−8^) and the CE/TC ratio (p-value < p_ratio_) are colored in red for log_10_(p-gain) > 11.2 and blue for ratio log_10_(p-gain) < 11.2.

We then asked whether any of the protein QTLs from the UKBPPP Olink study were overlapping with the CE/TC gene loci and found an intriguingly strong positive correlation between the effect sizes of the genetic associations with blood circulating LDL receptor levels (LDLR) and the CE/TC ratio (Figure 5). In 45 cases genetically perturbing CE/TC and LDLR leads to significant and comparable effects. The only genetic locus where the direction of the association with CE/TC and with LDLR is significant but opposite is ABCA6 (rs77542162). At two locations, SLC22A1 (rs2297359) and LPA (rs10455872), a significant association with CE/TC was found but not paralleled by an association with LDLR. As LPA represents an entirely different lipoprotein type that does not interact with LDLR, this would be expected.

Taken together, these associations suggest that the genes identified here in association with the CE/TC ratio have an impact on the homeostasis of fatty acyl groups attached to cholesterol moieties, be it directly by regulating enzymatic reactions that remodel the fatty acid side chains of lipids contained in the lipoproteins, or indirectly through control of lipoprotein transport. Their effect is in one direction for HDL and VLDL particles (except for small VLDLs) and in the opposite direction for LDL particles, suggesting that genetically induced changes in cholesteryl ester content may potentially be counter-balanced by the abundance of blood circulating LDLR, which is most likely present in its cleaved soluble form (sLDLR) and has been suggested to suppress binding of cell bound LDLR to APOB^31,32^. Alternatively, changes in CE/TC may also induce compensatory changes in LDLR expression. More research is needed to answer these questions.

A side note regarding the statistics of using ratios: the association of LDLR with CE/TC exemplifies the concept that using ratios can drastically improve association statistics where one trait may account for variance shared with the other (see R-output in Figure S4). In this case, 33.7% of the variance in LDLR can be explained by the cholesteryl ester percentage, while cholesteryl ester or total LDL cholesterol alone only explain 2.3% or 3.6%, respectively. Interestingly, the effect sizes of the association of the linear combination of CE and TC with LDLR are almost identical but with opposite directionality. As these traits are on a logarithmic scale, this implies that the ratio of both variables best describes the observed relationship with LDLR levels, since log (LDLR) ~ log (CE) – log(TC) = log (CE/TC).

### The percentage of HDL particles improves the prediction of incident myocardial infarctions.

**Figure 6:**
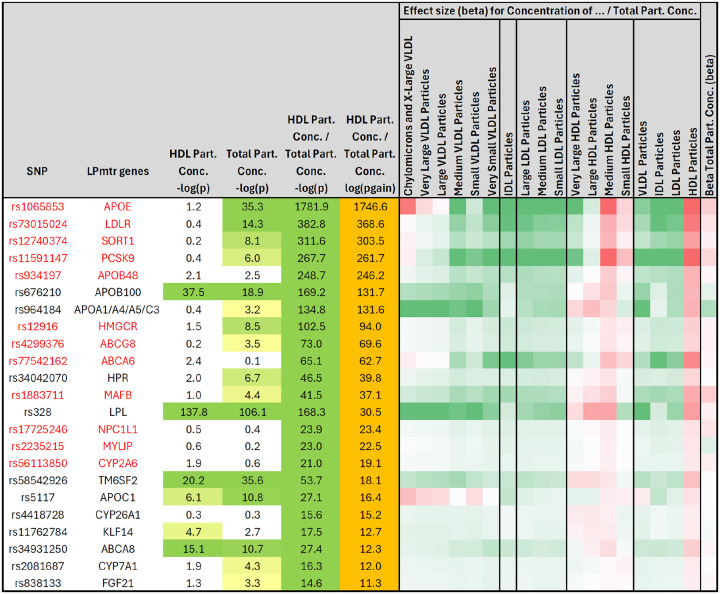
Heatmap of the effect sizes of the genetic associations with HDL particle concentration divided by total lipoprotein particle concentration in the different lipoprotein size classes. P-values and p-gains for all significant associations (p-gain > 10^11.2^) of all LPmtr gene loci with the ratio of the HDL particle concentration divided by the total lipoprotein particle concentration (left); Effect sizes for the associations of the particle number concentration of the individual specific lipoprotein size classes and types divided by the total particle concentration and of the total particle concentration alone (right); The effect directionality is represented relative to the directionality of the ratio of HDL particles dived by total particle concentration (plot data is in Table S11); Genes that are part of the HDL particle concentration divided by total lipoprotein particle concentration cluster are in red.

The second largest cluster is represented by the *Concentration of HDL Particles / Total Concentration of Lipoprotein Particles* ratio, which corresponds to the percentage of lipoprotein particles that fall into the HDL size classes (HDL-P %). HDL particles are the smallest and by number the most abundant lipoproteins. The highest p-gain is observed for the association with APOE (rs1065853) where the association with the individual traits is only moderately significant (p = 10^−35^) while the association with HDL-P % increases by over 1,700 orders of magnitude (p = 10^−1746^) (Figure 6 and boxplots in Figure 3D–F). Comparing the genetic associations with different size-classes suggests that genetic variance, especially at the APOA1/A4/A5/C3 and the LPL locus, is modulating the particle number percentage of larger VLDL particles in a direction opposite to its effect on HDL particles, with the markable exception of the APOE locus, where the genetic variant has an effect in the same direction on chylomicrons and very large VLDL particles as it has on HDL particles. While it is beyond the scope of our present work to speculate about the mechanistic drivers behind these observations, these examples demonstrate how associations with NMR traits and their ratios can provide new insights into the specific effects of selected genetic variants of interest.

**Figure 7:**
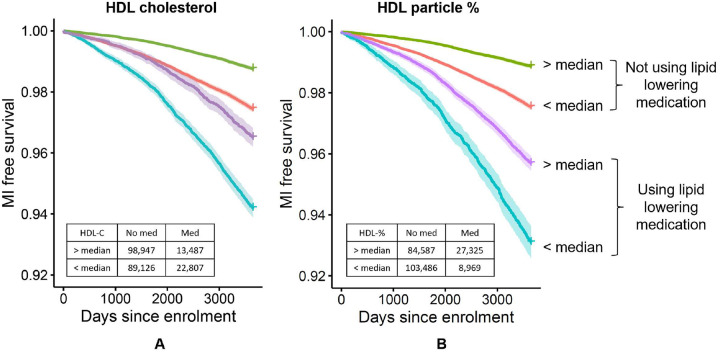
Survival curves for incident myocardial infarction. Ten-year survival analysis for incident myocardial infarction; Study participants were split into four groups, individuals taking or not taking lipid lowering medication and those with high/low HDL cholesterol (A) or ratio of the HDL particle concentration divided by the total particle concentration (B), where high is considered a value above the cohort median; Individuals with prevalent MI were excluded; The number of individuals per group reported on the plots and the 95% confidence intervals are shown.

A survival analysis with the ten-year incidence of MI suggests that HDL-P % is a better predictor than HDL-C: a person using lipid lowering medication has a 5.81% [5.51–6.12] risk of suffering an MI within the next ten year if their HDL-C is below the cohort-wide median while they have a 6.90% [6.38–7.42] risk if their HDL-P % is below its population median (Figure 7). This observation agrees with a clinical trial on the effect of statins on CVD that found HDL particle number to be a better marker of residual risk than chemically measured HDL-C^33^. HDL-P % may thus represent a biochemically more closely related marker for MI risk than HDL-C and can be derived from NMR lipoprotein profiling in a clinical setting.

From a functional perspective it is noteworthy that the genes that were associated at large p-gains with HDL-P % share a similar transcriptional repertoire, being controlled by SREBPs and LXRs, and most of them are involved in mechanisms related to LDLR mediated LDL-C uptake and cholesterol transport. In this context it is also interesting to note that the genes coding for the targets of three of the most successful cholesterol-lowering drugs (statins:*HMGCR*, ezetimibe:*NPC1L1*, and evolucumab:*PCSK9*) all associate with a significant p-gain with this ratio. Taken together, these observations support the idea that shared associations with ratios may reveal genes that are linked by shared biological processes, suggesting similar analyses for the other ratios and clusters we report here.

### Refined metabolic traits profile links miR-148 locus to coordinated hepatic lipid flux

Finally, we asked whether our data could guide us to generate new hypotheses for experimental follow-up while potentially uncovering novel regulatory pathways. Previous GWAS implied an association between *MIR148A* locus and blood TG and non-HDL-C levels^34^. However, while downstream analysis by us and others revealed a link between miR-148a and LDL-uptake through its direct inhibition of LDLR^35,36^, a functional connection explaining how miR-148a affects TG levels as its lead association remains obscure.

**Figure 8.**
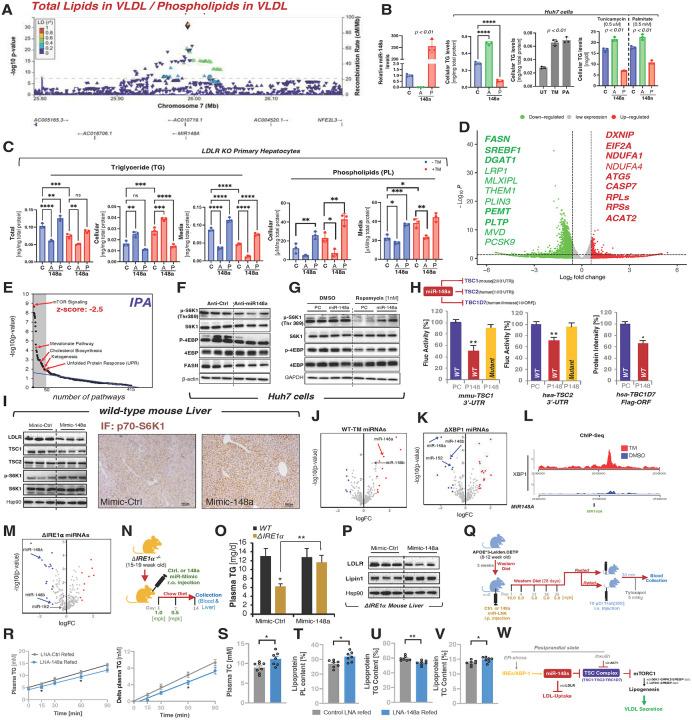
MiR-148a regulates VLDL lipid core composition and secretion. **(A)** Locus plot showing associations of phospholipid (PL) percentage in VLDL particles at the region harboring miR-148a; (**B**) Changes in miR-148–3p level sin Huh levesl alteres Cellular TG level in absence and presence of palmiate(PM or Tunicmaycin (TM) induced Er-Stress (**C**) TG secretion asssay performed in isolated LDLR deficient mouse primary hepatocytes in response to modulated miR-148a levels in the presernce and absence of ER-stress. C ellular and media TG and PL level are shown. (**D**) Volcano plot of differential gene expression in LNA-induced miR148a depleted Huh7 cells versus control LNA treated cells; (**E**) Ingenuity Pathway analysis (IPA) showing enrichment for genes involved in mTOR signaling and lipid metabolism following miR-148a repression in Huh7 hepatoma cells; (**F**) Immunoblot analysis of mTORC1 substrates in response to LNA mediated miR-148a inhibition after 36 hours in Huh7 cells with β-actin used as loading control; **(G)** Immunoblot analysis of mTORC1 activity in response to miR-148a expression after 36 of transfection in the presence and absence of Rapamycin (1mM) treatment for 4 h with GAPDH used as a loading control; (**H**) Validation of predicted 148a targets TSC1, TSC2 and TBC1D7, three major components of the TSC complex; Bar blots show miR148a luciferase activity in Huh7 cells transfected with a FLuc reporter containing wild-type or mutated miR-148a binding site of mouse TCS1 and human *TSC2* 3’-UTR, co-transfected with the indicated miRNA precursors; Ectopic expression of flag-tagged human TBC1D7 protein with wild-type (WT) miR-148a-3p binding site; TBC1D7 constructs were co-transfected with precursor miR-control (PC), miR-148a-3p (P148a); (**I**) Expression levels of miR-148a targets LDLR and TSC1 and its downstream effect on mTORC1 activity in wildtype mice treated with control and miR-148a mimics and immunofluorescence staining of the phosphorylated form of S6K1 in prepared liver sections from mice in response to miR148a treatment; (**J-K**) Volcano plot representation of the differentially expressed miRNAs from wildtype mice treated with TM and from XBP1-LKO mice compared to wild-type littermates; **(L)** XBP1 binding peak approximately 1.5k bases upstream of MIR148a gene in control and TM treated T47D breast cancer cells; (**M**) Volcano plot depiction of altered hepatic microRNA profile of IRE1-LKO mice; Arrows highlight downregulated miR148a and its isoforms; (**N**) Schematic representation of miR-148a mimic treatment strategy of hypotriglyceridemic IRE1a deficient mice; (**O)** Altered plasma triglyceride levels measured in IRE1α KO mice following miR-148a injection as compared to control mimic; **(P)** Protein analysis of hepatic LDLR and Lipin1 expression in mice injected with miR-148a (*n* = 3–4 per group); HSP90 was used as a loading control; **(Q)** Schematic representation of the LNA-mediated miR-148a loss of function experiment in hyperlipidemic ApoE*3-Leiden using CETP transgenic mice for VLDL secretion analysis at fasted and postprandial state; (**R**) Time-resolved analysis of reduced hepatic VLDL-TG secretion in response to miR-148a inhibition; (**S**) miR-148a depletion increases plasma TC levels; (**T-V**) Altered lipid core composition of isolated plasma VLDL particles upon secretion from liver in response to miR-148a repression; (**W**) Proposed cellular mechanism of coordinated regulation of LDL-uptake and VLDL-secretion by miR-148a at the postprandial state. Statistical significance between groups was calculated by one-way ANOVA. *P < 0.05, **P < 0.01, ***P < 0.001, ****P < 0.0001. Bar graphs represent means ± SD.

Graham *et al*. identified seven variants at the *MIR148A* locus associated with lipid traits, highlighting three independent genetic signals (Table S3), with rs4722551 displaying the strongest association with LDL-C, TG, and non-HDL-C. Notably, while replication in our dataset narrowly missed genome-wide significance for TG (p = 2.1×10^−8^), NMR-based lipidomics uncovered multiple Bonferroni-significant associations, most prominently with free cholesterol in chylomicrons and extremely large VLDL particles (p = 1.4×10^−13^). Further ratio analyses identified a strong association of the *MIR148A* locus with the VLDL total lipid-to-phospholipid ratio – a proxy for VLDL phospholipid content (Figure 8A).

Phospholipids (PLs) are essential for VLDL particle enlargement and stability; reduced PL content has been linked to impaired TG-rich VLDL secretion.^37,38^. This process is modulated by intracellular phospholipid transfer protein (PLTP), which promotes VLDL expansion, while circulating PLTP transfers PLs to HDL, thus reducing VLDL size during lipolysis. Notably, we detected no association between PLTP and rs4722551 (p = 0.783, UKBPPP Olink), nor any predicted miR-148a binding sites in PLTP’s 3′UTR. These observations suggest that miR-148a may influence VLDL phospholipid content at the intracellular level rather than in circulation, particularly by affecting PC phosphatidylcholine (PC) synthesis and the PC/PE (phosphatidylcholine/phosphatidylethanolamine) ratio that governs ER membrane curvature and fluidity and thus ER-stress, the disruption of which shifts the equilibrium from TG secretion to lipid storage, driving steatosis^39^ and changing plasma VLDL composition^39,40^.

These preliminary observations led us to hypothesize a role for miR-148 in VLDL assembly and secretion, potentially as part of a coordinated regulatory mechanism that acts to limit VLDL/LDL recapture via LDLR inhibition, while promoting TG export into plasma.

To assess miR-148a function in TG–VLDL metabolism, we first performed a series of *in vitro* and *ex vivo* TG secretion assays and quantified cellular and media TG levels following experimental manipulation of miR-148a expression using precursor constructs to elevate, or antisense oligonucleotides to suppress, miR-148a levels (Figure 8B). Increasing miR-148a reduced cellular TG content, whereas antisense-mediated depletion markedly elevated TG levels in Huh7 cells. Transient overexpression of miR-148a consistently lowered cellular TG levels, whereas antisense-mediated inhibition produced a robust increase in TG accumulation in Huh7 cells. Under ER-stress conditions induced by tunicamycin (TM) or palmitate (PM), both of which elevate intracellular TG due to enhanced lipogenesis (DNL) and and impaired VLDL secretion (Fig 8B), loss of miR-148a exacerbated TG accumulation, while miR-148a overexpression significantly attenuated it.

Hepatic carsinoma cells lines exhibit intrinsically high TG levels due to increases DNL and reduced TG secretion and β-oxidation capacities relative to primary hepatocytes. Therefore, we next assessed miR-148a function on TG secretion in isolated primary liver cells. Thereby we choose LDLR-deficient primary hepatocytes to exclude effects secondary to miR-148a–mediated LDLR repression^40,41^. miR-148a depletion increased cellular TG while reducing media TG levels, whereas transient miR-148a overexpression lowered intracellular TG and increased secreted TG, indicating enhanced VLDL output. These effects persisted under tunicamycin-induced ER stress, underscoring a protective role for miR-148a in maintaining VLDL-secretion capacity. Summed cellular and media TG levels a measure for total TG levels further showed that miR-148a loss impairs both VLDL biogenesis and secretion, defects that were reversed upon miR-148a overexpression (Figure 8C).

Cellular PL metabolism plays a decisive role in early stage VLDL lipidation process^40,42^. Intriguingly, antisense-mediated miR-148a loss significantly reduced total PL, whereas miR-148a elevation increased PL abundance (Figure 8C). These findings parallel the ratio-based association between miR-148a and PL composition of VLDL particles (Figures 8A, 8C) and highlight a broader role for miR-148a in VLDL assembly.

Since miR-148a is among the most highly expressed microRNAs in the liver, we first sought to gain insights into the impact of intrinsic miR-148a expression on key liver metabolic functions. We identified transcriptome-wide changes in gene expression profiles by RNA-sequencing in response to antisense-mediated miR-148a depletion (Figure 8D), that were associated with reduced lipogenesis (e.g. *FASN, SREBF1, DGAT1, CHREBP and THEM1*), PC/PE ratio and VLDL size (*PEMT, PLTP*), increased ER-stress (*EIF2 and the* direct miR-148a target gene *DXNIP*) and members of ribosomal proteins (*RPLs and RPSs*), reflecting reduced protein synthesis. When combined with an unbiased bioinformatic approach such as IPA, we identified the central nutrient and energy sensing mTOR pathway (−log10p= 8.76, z-score=−2.5) as one of the most significantly altered pathways beside major metabolic pathways in lipid/cholesterol metabolism (Figure 8E). Conversely, miR-148a overexpression led to an increase in the mTOR signaling pathway with a z-score of 2 (data not shown). This intriguing observation prompted further investigation into the role of miR-148a in modulating the mTOR pathway.

At the protein level, miR-148a antagonism in cells resulted in a reduction in phosphorylated 70S6 kinase 1 (p70S6k1) and eukaryotic initiation factor 4E-binding protein 1 (p4E-BP1) protein abundance, two major downstream events specific to mTORC1 signaling that control SREBP-dependent lipogenesis and cap-dependent translation, respectively (Figure 8F). In accord, we obtained a significant reduction in fatty acid synthase (FASN, a canonical SREBP1c target gene that increases the synthesis of triglycerides in liver, at both protein (Figure 8F) and mRNA level (Figure 8D). Conversely, in response to rapamycin-induced inhibition of mTORC1, miR-148a mimic-mediated overexpression sustained mTORC1 activity reflected by relatively higher p70S6K and p4E-BP1 protein levels, suggesting a role for miR-148a in mTORC1 activation (Figure 8G).

During the postprandial state, the surge in nutrients and subsequent elevated insulin levels instigate extensive metabolic changes in the liver, thereby promoting mTORC1 activity and downstream transcriptional program towards increased lipogenesis and glycolysis, aside from protein translation. This involves predominantly upstream AKT1-mediated inhibition of TSC complex leading to Rheb-dependent inhibition of mTORC1 activity. Intriguingly, bioinformatics analyses revealed that miR-148a targets all three principal components of the TSC complex – TSC1, TSC2, and TBC1D7 – for post-transcriptional repression. In primates, binding sites reside within the 3′ untranslated regions (3′UTRs) of TSC2, while in rodents, they are found in the 3′UTRs of TSC1. However, TBC1D7 contains a conserved miR-148a binding site within its coding region, shared across both primate and rodent lineages (Figure 8H and S7a–c).

Consistent with this prediction, the transient expression of miR-148a led to a significant reduction in both the mRNA and protein levels of TSC2 and TBC1D7 in the Huh7 cell (Figure S7d–f). Unexpectedly, TSC1 was also reduced, likely via destabilization secondary to TSC2 repression. To validate the direct binding of miR-148a to the 3’ UTR regions of human TSC2, and mouse TSC1, we performed 3’ UTR luciferase reporter assays. The results demonstrated that transient expression of miR-148a significantly reduced luciferase activity relative to control (Figure S7f). Importantly, site-specific mutation within the miR-148a binding sites abolished the inhibitory effect of miR-148a on the luciferase activity of these target genes (Figure 8H and S7a–c). To verify miR-148a’s direct inhibitory effect on TBC1D7, a Flag-tagged construct harboring human TBC1D7 cDNA was generated, and its expression assessed at the protein level in Huh7 cells. As expected, mimic-induced miR-148a expression resulted in a significant reduction of TBC1D7 protein levels (Figure 8H and S7d).

To show that miR-148a effect on mTORC1 activity is dependent on TSC, we performed antisense-mediated inhibition of miR-148a in wild type and TSC2-deficient mouse embryonic fibroblasts (MEFs). We observed that while miR-148a knockdown significantly reduces 4EBP1 phosphorylation in TSC2+/+ cells, it had no effect in TSC2−/− cells. Additionally, miR-148a knockdown increased TSC1 expression only in TSC2+/+ cells, but not in TSC2−/− cells. We speculate that the induction of TSC1 by miR-148a depletion requires the intact components of TSC complex (Figure S12a&b).

To test miR-148a trans-activating effect on mTORC1 activity we performed a gain of function experiment in wild-type C57BL/6J mice injected with liver-targeting miR-148a mimics. Thereby, the miR-148a mimic treated liver displayed enhanced S6K1 phosphorylation as assessed by analysis of liver protein levels and immunofluorescence staining of liver sections for p70S6K1. We confirmed that miR-148a mimics repressed both TSC1 and the previously identified direct target LDLR (Figure 8I).

The transactivation of the mTORC1 signaling by miR-148a raised questions regarding the upstream regulation of this microRNA. Given mTORC1 role in lipogenesis and concomitant metabolic stress caused at the postprandial state in part due to lipid overload, we considered the endoplasmic reticulum (ER) stress pathways also known as unfolded protein response (UPR), as a plausible upstream signal influencing its expression. To explore miRNA responses to ER stress, we profiled mouse livers treated with ER-stress inducer tunicamycin (TM) and found a marked induction of miR-148a and its isoform miR-148b as compared to untreated controls (Figure 8J). Moreover, miR-148a expression were positively affected by palmitate in Huh7 cells in a dose dependent manner (Figure S8). A similar trend was found in the liver of major mouse models of obesity (*ob/ob* and *db/db* mice) with chronic ER-stress reflected in increased GRP78, CHOP, and XBP1s expression, further underscoring the metabolic relevance of miR-148a during ER-stress (Figure S8).

To examine whether the IRE1a-XBP1 regulatory arm of UPR that has an established role in hepatic lipogenesis and VLDL assembly and secretion transcriptionally increases miR-148a expression in response to ER-stress, we conducted a global analysis of all microRNAs in the liver of mice with liver-specific XBP1 deficiency^43^. As shown in Figure 8K, the level of miR-148a and its other two isoforms were markedly reduced in the absence of XBP1 and remained unaffected in XBP1 KO mice treated with TM (Figure S8c). Conversely, liver-targeted adenoviral overexpression of spliced active form of XBP1 upregulated hepatic expression of miR-148a (~4–5 fold) and miR-148b (~2 fold) (Figure S8d), suggesting that hepatic miR-148a is a direct transcriptional target of XBP1. Indeed, ChIP-Seq data analysis revealed that spliced XBP1 binds to the upstream region of the *MIR148A* and its isoforms host genes *COPZ1* and *COPZ2* under ER-Stress conditions in T47D breast cancer cells (Figure 8L and S10a&b). Similarly, XBP1 binding at miR-148a/b locus was verified in multiple ChIP assays performed in Huh7 and HepG2 cells (**Figure 10c-i**).

Next, we examined miR-148a regulatory function in mTORC1-mediated lipogenesis in response to ER-stress in Huh7 cells. Consistent with ER stress–induced activation of lipogenesis, cells treated with TM exhibited an increased level of selected key enzymes involved in fatty acid/TG synthesis such as FASN and SCD1 (Figure S11a). However, despite a modest but significant decrease in lipogenic gene expression in the absence of ER-stress, LNA-mediated repression of cell-intrinsic miR-148a levels blunted the increase in lipogenic gene expression in the presence of TM-induced ER-stress (Figure S11a). These results suggest that while miR-148a inhibition suppresses mTORC1 activity under basal conditions, explained by direct inhibition of the TSC complex, enhanced XBP1-induced miR-148a expression under ER-stress might coordinately act with XBP1s to expand ER-biogenesis and capacity by activating mTORC1-mediated lipogenesis and promoting VLDL secretion to restore ER-function.

To show that miR-148a restores VLDL-secretion in states of chronic ER-stress and hepatic steatosis independent of regulated IRE1α-dependent decay (RIDD), we chose to restore compromised miR-148a levels in IRE1α-deficient mice that displayed significantly reduced miR-148a levels (Figure 8M). Strikingly, we observed that miR-148a treatment rescued the hypotriglyceridemic state in IRE1α-deficient mice by elevating plasma TG levels in accompany with a border-significant decrease in hepatic TG levels (Figure 8N, M and S11c). Thereby, the total plasma cholesterol levels remained unchanged (Figure S11b). LDLR expression was strongly reduced by the expression of miR-148a in IRE1α KO mice, while we observed a significant increase in Lipin1 protein levels, a SREBP1c target gene promoting TG synthesis and VLDL secretion in liver in addition to its activity being regulated by mTORC1 (Figure 8P)^44,45^.

To further validate the functional role of miR-148a in regulating hepatic triglyceride output and VLDL composition, we examined its impact on postprandial VLDL production in the APOE*3-Leiden.CETP model (Figure 8Q), in which the lipoprotein content consists mainly of VLDL particles and hepatic miR-148a levels are significantly higher by 3–4-fold (data not shown). Assessment of VLDL production in the postprandial state showed a reduction in VLDL-TG secretion in mice treated with miR-148a LNAs compared to control LNAs (Figure 8R), without affecting VLDL-ApoB output (data not shown). Analysis of the VLDL fractions, isolated after tyloxapol administration, showed that miR-148a-LNA treated mice displayed reduced VLDL-associated TG and increased PL content (Figure 8T and U). Thereby total cholesterol content in the lipoprotein fraction and plasma were increased (Figure 8S&V) accompanied by a trend toward smaller VLDL particle size (data not shown). These findings demonstrate that miR-148a affects VLDL-PL and TG composition and overall lipoprotein lipid distribution.

Taken together, our findings highlight miR-148a as a key regulatory node integrating ER stress, mTORC1 signaling, and lipoprotein metabolism. Through repression of LDLR and the TSC complex, miR-148a promotes SREBP-driven lipogenesis and VLDL secretion while simultaneously limiting LDL uptake in the postprandial state, thereby coordinating hepatic lipid efflux during the postprandial state (Figure 8W).

### Ratio profile associations reveal functional links among co-clustered lipid loci

**Figure 9.**
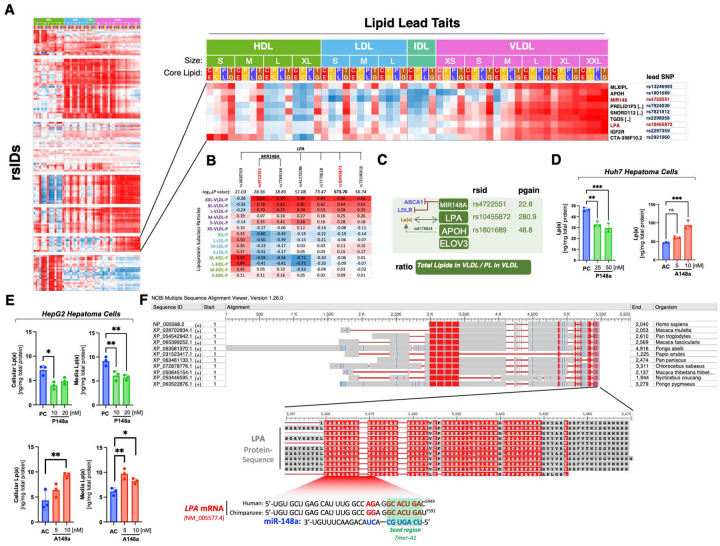
Shared ratio profile associations links miR-148a to Lp(a) regulation. **(A)** Heatmap of lipoprotein subclass and their lipid core associations highlighting effects of SNPs across lipid traits. The zoomed cluster contains the miR-148a locus. **(B)** Strong correlation of *MIR148a* and *LPA* association patterns across all their variants. (**C**) *MIR148A* and *LPA* loci joint association with VLDL total lipid-to-phospholipid ratio. (**D**-**E**) Altered Lp(a) levels in response to concentration dependent miR-148a antisense or mimic mediated alterations measured in Huh7(D) and HepG2 cells as indicated (AC: antisense control, PC: precursor control). (**F**) Alignment of *LPA* protein sequences from human and non-human primates highlighting regions of high conservation (red). The putative miR-148a interaction site is located within the coding region (ORF) in close proximity to the 3′-UTR. Statistical significance between groups was calculated by one-way ANOVA. *P < 0.05, **P < 0.01, ***P < 0.001, ****P < 0.0001. Bar graphs represent means ± SD.

While NMR lipid profiles highlight common loci with shared biology, ratio-driven associations can provide a deeper picture of potential functional links among co-clustered gene variants that previously remained unrecognized. We found that *MIR148A* and *LPA* variants show similar association patterns across the lipoprotein measures (Figure 9A and B). Moreover, the miR-148a associated lipid ratio trait was also enriched at the *APOH*, *ELOVL3*, and *LPA* loci (Figure 3C, 9C, Table S8), suggesting a potential functional association/interaction between the corresponding genes. Notably, this joint association was supported by a recent study demonstrating and genetic variant in *APOH* increases LPA expression^46^.

To determine whether miR-148a also influences Lp(a) production, we quantified secreted Lp(a) levels using a highly sensitive ELISA validated to exclude apoB and plasminogen cross-reactivity. Manipulation of miR-148a expression in vitro revealed a reciprocal relationship: antisense-mediated depletion of miR-148a increased Lp(a) levels in the culture media (Figure 9D and E), whereas miR-148a overexpression reduced Lp(a) secretion in both Huh7 and HepG2 cells, similarly, to apo(a) protein levels measured in cells (Figure 9D and E). These findings indicate that miR-148a exerts a direct inhibitory effect on Lp(a) output.

In silico analysis of the human *LPA* transcript (NM_005577.4) across multiple miRNA– target prediction platforms did not identify high-confidence canonical 7–8-mer miR-148a-3p binding sites within the annotated 3′-UTR, and *LPA* is not listed among validated miR-148a targets in curated databases. These findings argue against established post-transcriptional repression of *LPA* by miR-148a. However, sequence scanning of the full *LPA* mRNA using canonical seed-matching criteria revealed a conserved 7mer-A1 motif in primates located near the 3′UTR region (Figure 9F), This putative site suggests the possibility of a previously unrecognized direct interaction through which miR-148a may influence Lp(a) metabolism through direct inhibition.

## DISCUSSION

Understanding the pathobiology of lipoproteins is arguably key to the development of new targeted therapies, but understanding the atherogenic potential of their lipidomic and proteomic composition is very much a ‘work-in-progress’^1^. Here we add new insights to this challenge by conducting a deep molecular phenotypic fine mapping of the Graham *et al*. lipid risk variants by generating biological insights at over 550 independent gene loci. At over half of these loci, the association strength significantly increased by using an NMR trait compared to using only one of the five “classical” lipid traits, indicating that for each of these loci additional biochemical information has been generated, arguably drawing closer to the true biology of the respective genes and loci. Moreover, in over two thirds of the cases, ratios between NMR traits further increased the strength of association at a genome-wide significance level and beyond, in many cases suggesting shared biology between the associated genes.

The lipoprotein triglyceride-, phospholipid- and cholesterol-content varies largely between individuals due to differences in genetics, lifestyle and nutritional preferences, inducing strong correlations between many of the NMR traits investigated here. By analyzing ratios, we account for the resulting variance that is shared between the individual traits, allowing us to identify genetic association signals that would otherwise be overwhelmed by noise.

We argue that genes that share an association with a same ratio at high p-gains are impacted by a same driver of shared variance and therefore may be linked by a same biological (or technical) confounder. In the case of the LA / PUFA ratio, this confounder may be a preference for PUFA-rich foods, and in the case of the CE / TC ratio a preference for food high in cholesterol. This hypothesis is supported for instance by the observation that many of the genes associated with the LA / PUFA ratio are indeed acting on connected biochemical pathways, like the phospholipases PLAG2G10, PLCH2, PNPLA3 and the mono- and diacylglycerol acyltransferases MOGAT and DGAT2. In the case of the CE / TC ratios, many of the associated genes control or modify the cholesteryl ester content of lipoprotein particles, and the effect of genetic variance in these genes reveals their role once the variance in total cholesterol is accounted for by the ratio. In addition, we found that the effect of genetic variance on the CE / TC ratio correlates with blood circulating LDLR protein levels and explains more than 30% of its variance.

Our follow-up investigation identified a novel regulatory extension of the canonical UPR branch, wherein miR-148a mediates ER-stress-induced lipogenesis, which genomic locus of which we found to be significantly associated with VLDL particle composition. This is an intriguing new defensive mechanism that appears to operate as an adaptive response contributing to XBP1-driven hepatic lipid production and secretion, facilitating ER membrane expansion under conditions of metabolic stress^47^ and thereby mitigating hepatic steatosis^48^. We speculate that, analogues to the impaired hepatic XBP1 function^49,50^ observed in metabolic disorders, restoration of miR-148a expression under conditions of chronic, un-resolved ER-stress such as obesity and NAFLD/NASH, may present a promising therapeutic strategy to counteract ER-dysfunction, as also proposed by others^51^.

In addition to the well-characterized insulin-mediated inhibition of the TSC complex, our findings reveal a novel role for miR-148a in modulating mTORC1 signaling through direct repression of key components of the TSC complex^52^. We propose that miR-148a functions as a central post-transcriptional regulator of hepatic lipid flux by coordinating both LDL-uptake and VLDL-secretion in the postprandial state.

Elevated Lp(a) is increasingly recognized as a major contributor to residual cardiovascular risk, even in individuals achieving optimal LDL-lowering with contemporary therapies. This unmet clinical need has positioned Lp(a) as a transformative target in precision cardiometabolic medicine, with the potential to meaningfully reduce global cardiovascular morbidity and mortality. Yet the molecular underpinnings that govern Lp(a) synthesis, assembly, and clearance remain incompletely resolved. Our findings introduce a miR-148a–directed post-transcriptional regulatory axis as a previously unknown contributor to Lp(a) biology. Although preliminary, these observations suggest that miR-148a may influence apo(a) expression or particle assembly and thus modulate circulating Lp(a) levels. Definitive mechanistic insight will require rigorous target-validation studies and functional manipulation of miR-148a in LPA-transgenic mouse models to establish causality and map the molecular circuitry underlying this regulatory relationship.

Our study has of course also its limitations, such as choices that had to be made regarding the scaling of the traits, the covariates used in the models, significance cut-offs etc. We followed as closely as possible previous work (i.e. Graham *et al*.), while being as conservative as possible (i.e. always using Bonferroni correction and not analyzing rare variants).

The validity of the Nightingale platform is critical to our study. While the NMR platform has been used in many previous studies^11,25,53^, we additionally confirmed that results obtained using the NMR platform concord with those obtained using clinical biochemistry. We found that the Pearson correlation between the readouts from both platforms was larger than r^2^ = 0.77 (Figure S2 A–E) and that the correlation between the effect sizes for the 274 lead associations was r^2^ = 0.981. Furthermore, the correlation between the effect sizes obtained using ~231,000 UKB samples and those reported by Graham et al. were r^2^ = 0.945 when using the NMR data and r^2^ = 0.960 when using the clinical biochemistry data (Figure S2 F–H). As we could not discuss all associations to the degree they deserved we share the complete association data in different formats and degrees of condensation as Supplementary Data on Figshare (https://doi.org/10.6084/m9.figshare.19728991). While it might be interesting to investigate possible causality between CE / TC and circulating LDLR levels, Mendelian randomization approaches did not appear appropriate here as the assumption of absence of horizontal pleiotropy is not valid for the highly correlated lipid traits.

Taken together, our deep molecular fine mapping of the Graham *et al*. lipid risk loci provides a comprehensive resource that can now be used to support the development of future lipid regulating drugs and treatment options. Most striking are the many direct biological links that could be identified between the lipoprotein and metabolic traits and ratios and their associated gene variants that modulate many disease-relevant processes of lipoproteins metabolism, transport, and remodeling. We established a clear role for 84 genes that play a potentially causal role in lipoprotein metabolism, transport, and remodeling. More generally, as we have shown at examples, our data can now be used to transfer knowledge from known to new lipid risk loci based on similarity of their respective NMR-trait and -ratio association profiles.

## METHODS

### Data sources.

All data was obtained through the UKB RAP system on the DNAnexus platform (data dispensed on August 11, 2023; application ids 43418 and 588633). Samples were restricted to baseline by requiring “Spectrometer |Instance 0” is not NULL, yielding 274,359 records with NMR data out of a total of 502,364 UKB participants. Samples were then split into a discovery and a replication set based on the variable “Genetic ethnic grouping”. The discovery set comprised 231,145 records for which “Genetic ethnic grouping” was equal to “Caucasian”. All other samples were assigned to the replication set and contained 43,214 records. Imputed genotypes for 1,835 variants were extracted from UK RAP BGEN files (https://biobank.ndph.ox.ac.uk/showcase/label.cgi?id=100319) using bgenix^54^ and reformatted to text format using plink^55^. NMR data (https://biobank.ndph.ox.ac.uk/showcase/label.cgi?id=220) and additional phenotype data (age, sex, use of cholesterol lowering medication, medication usage, clinical biochemistry, …) were extracted using the DNAnexus cohort browser and the table downloader app (https://ukbiobank.dnanexus.com/landing). Incident myocardial infarction was defined as present when the reported “Date of myocardial infarction” (https://biobank.ndph.ox.ac.uk/showcase/field.cgi?id=42000) was later than the “Date of attending assessment centre” (https://biobank.ndph.ox.ac.uk/ukb/field.cgi?id=53). For all variables for which multiple instances were available, “Instance 0” (baseline) was selected.

### Lipoprotein and metabolic data.

A detailed description of the metabolic traits and pathways covered by the Nightingale NMR platform is provided in Table S1 and https://biobank.ndph.ox.ac.uk/showcase/label.cgi?id=220. In brief, the platform readouts include several amino acids (alanine, glutamine, glycine, histidine, isoleucine, leucine, valine, phenylalanine, tyrosine), glycolysis related metabolites (glucose, lactate, pyruvate, citrate), ketone bodies (3-hydroxybutyrate, acetate, acetoacetate, acetone), creatinine, and two lipid species (linoleic acid, docosahexaenoic acid). The Nightingale platform further reports aggregated lipid traits, specifically, total fatty acids and their degree of unsaturation, omega-3 and omega-6 fatty acids, polyunsaturated fatty acids (PUFA), monounsaturated fatty acids (MUFAs), saturated fatty acids, phosphoglycerates, total cholines, phosphatidylcholines (PCs), and sphingomyelins (SMs). Moreover, the platform also provides readouts of protein glycosylation (glycoprotein acetyls), apolipoproteins B (ApoB) and A1 (ApoA1), and albumin. However, the largest part of the platform’s traits are quantitative measures of total lipids, free and total cholesterol (FC, TC), cholesteryl esters (CE), phospholipids (PL), and triglycerides (TGs), all segregated by lipid particle size, together with the concentrations of these particles. Lipoprotein particle classes cover fourteen size ranges, from small HDL particles with an average diameter of 8.7 nm to chylomicrons and extremely large VLDL, with particle diameters from 75 nm upwards. In addition to the individual traits, Nightingale suggests the computation of 81 ratios between these directly measured traits as additional endpoints, including the ratios of TGs to phosphoglycerides, apoB to apoA1, PUFAs to MUFAs, omega-6 fatty acids to omega-3 fatty acids, and the percentages of different lipid content in the lipoprotein size classes, bringing the total number of features up to 249 (Table S2).

### Statistical analysis.

The NMR data was log-scaled and ratios were computed using the identity log(A/B) = log(A) - log(B), which renders the association statistics invariant to inversion of nominator and denominator. NMR values equal to zero were treated as missing. The fraction of missing and zero values was very low, with 20,856 NA’s (0.045%) and 5,940 zero values (0.013%) out of 274,359*168 = 46,092,312 individual datapoints. The data was inverse-normal scaled after log-scaling to avoid overly strong effects of outliers that may have resulted from division by small values. Linear models were computed using R (version 4.1.0) using the R-package maplet (version 1.1.1)^56^, with the NMR data as dependent variables and all genetic variants reported in Supplementary Table 3 by Graham *et al*., plus age, age^2^, sex, and use of lipid lowering drugs to the model (binary) as independent variables. For ratios between two NMR traits, the p-gain was computed as the smaller of the two p-values for the individual trait associations divided by the p-value for the ratio^17^. Assuming a genome-wide multiple testing level of ~10^6^ tests and requiring nominal significance (p<0.05, p-gain>10 in case of ratios^17^), the following Bonferroni levels of significance were applied, depending on the context:

- p_ref_ = 5×10^−8^/5 = 10^−8^ for testing the five lipid traits (also used by Graham *et al*.),
- p_NMR_ = 5×10^−8^ / 168 = 10^−9.5^ for testing the NMR traits,
- p_NightRatios_ = 5×10^−8^ / 249 = 10^−9.7^ for testing NightRatios,
- p_AllRatios_ = 5×10^−8^ / (168*169/2) = 10^−11.5^ for testing all ratios & traits,
- p-gain_AllRatios_ = 10^6^ * 10 * (168*169/2) = 10^11.2^ for testing all ratios & traits.

### Associations with medication use and incident MI.

Incident MI was defined as true when the date of a reported MI was later than the date of the participant’s visit to the UKB assessment center. Covariates used in the model were age, age^2^, sex, HbA1c and glucose. In the associations with statins, individuals using cholesterol lowering drugs but not statins were excluded. Association with incident MI was limited to samples from individuals not using any cholesterol lowering medication. Summary statistics for the trait associations shown in Figures 2 and S3 are in Table S15.

### Survival analysis with incident MI.

Survival analysis of myocardial infarction was conducted over a time span of 10 years using the R package “survival” (version 3.3–1)^57^. Individuals with prevalent were excluded. The remaining samples were split at the median value into groups with high and low HDL-C and HDL-P % levels and then further into groups reporting the use of lipid lowering medication or not.

### Annotation of the LPmtr genes.

Using SNiPA^58^, GeneCards^59^, PhenoScanner^60^, and general PubMed searches, we identified 101 gene loci among the 554 variants associated with ratios that encode proteins involved in lipoprotein metabolism, transport, and remodeling (LPmtr genes), and that are – given their functions, associated NMR traits, and absence of alternative candidates – most likely causal for the observed lipid trait associations. We excluded genetic loci where the functional gene could not be readily identified or where the likely causal gene was functions not directly related to LPmtr, such as energy homeostasis, inflammation, and development. We assigned only the strongest variant at each locus to an LPmtr gene, excluding secondary genetic signals from the cluster analysis.

### Overlap with UKB-PPP protein pQTLs.

We identified the overlap of protein QTL data from the UKB-PPP project^61^ with the variants reported by Graham et al.. Association data were available for all but two of the 1,054 lead variants we analyzed here (rs368178 and rs448092) and are reported in Table S21 for all pQTLs that reached a significance level of 10^−8^ or above. The strongest pQTL for each variant is also reported in Table S3. The full summary statistics for UKB-PPP are available at http://ukb-ppp.gwas.eu.

### Functional variant annotation.

We used the Open Targets^30^ platform (version 22.10) via its API to annotate variant associations with the most likely causal genes, variant effect, overlapping disease GWAS hits, and gene expression, splice variant and proteomics QTLs in order of increasing p-value. The look-up comprises same variant across stored data sets and reference to GWAS regional lead signals using LD, limited to LD r^2^>0.7 between the Graham *et al*. and the GWAS lead signals (Table S3).

### Cell Culture.

Huh7or HepG2 cells were grown in DMEM supplemented with 10% fetal bovine serum with 1% penicillin-streptomycin and kept at 37 °C, 5% CO2. 130,000 cells were plated in 6 well plates and treated with tunicamycin (Sigma), tapsigargin (Calbiochem), or Brefeldin A (Cell Signaling Technology) for 12h.

### Mouse Experiments.

All experiments were performed in accordance the relevant animal welfare protocols and with the approval of the Central Authority for Scientific Procedures on Animals (CCD) of the Netherlands. Generation of XBP1 knockout mice has been previously described (Lee et al., 2008). Ern1 knockout mice were generated has been described previously (Hur et al., 2009). XBP1 WT, LSKO and ERN1 WT, LSKO received intraperitoneal (IP) injections of tunicamycin at a dose of 1 mg/kg body weight, control mice were treated with DMSO in phosphate-buffered saline (PBS) for 12 h. Male C57bl/6J, B6.cg-lepob/J, B6.BKS(D)-Leprdb/J mice were purchased at 8 weeks of age from Jackson Laboratory and housed three to five mice per cage with free access to water and normal chow diet. The mice being euthanized, and organs were collected at 12 weeks of age.

### In vivo miRNA mimic delivery and plasma lipid analysis.

Ire1α WT (n = 7) and KO (n = 7) mice 15- to 19-weeks old were subjected to a retro-orbital injection of control mimic (CTL mimic; n = 3 per genotype) or miR-148a mimic (1 mg/kg; n = 4 per genotype). Mice were allowed to recover for 7 days and then subjected to a second retro-orbital injection of control mimic or miR-148a mimic (0.5 mg/kg). After a 7-day recover period, mice were fasted for 6 hours and then blood and liver tissue samples were collected. Blood samples were processed to plasma, pooled, and then subjected to size exclusion chromatography to separate lipoproteins. Biochemical assays were used to measure plasma lipid levels.

### RNA isolation and qRT-PCR.

Total RNA was isolated from liver tissues using Trizol reagent (Thermo Fisher Scientific) and from Huh7 cells using miRNeasy kit (Qiagen). Reverse transcription was carried out with the iScript cDNA synthesis kit (Bio-Rad) using 500 ng of total RNAs. Quantitative real-time -PCR was performed using SYBR green (Thermo Fisher Scientific) on a ViiA^™^ Real time PCR system (Applied biosystems Inc). The miRNA cDNA was synthesized using the miRCURY LNA^™^ Universal RT microRNA PCR system. The cDNA template is then amplified using miRNA-specific LNA-based primers (Exiqon) and SYBR green was used for detection. Primer sequences used in this study are available upon request.

### RNA-seq (Ribo-Zero Paired-End analysis).

Total RNA (2 μg) was used for ribosomal RNA (rRNA) depletion using Ribo-Zero rRNA Removal Kit (Human/Mouse/Rat) and rRNA depletion was performed according to the manufactures protocol (Illumina). Briefly, 100 ng of rRNA depleted RNA was used to generate strand-specific libraries with BIOO NEXTflex Rapid Directional RNA-Seq Kit (Bioo-Scientific, Austin, TX) according to the manufacturer’s protocol. Library quality and quantity were analyzed with the Bio analyzer 2100 (Agilent, Santa Clara, CA) on a High Sensitivity DNA chip. The libraries were then normalized and pooled in equimolar ratios. Every 6 libraries were sequenced in a single lane of an Illumina HiSeq 4000 paired end 75bp run.

### Lp(a) secretion assay:

Huh7 and HepG2 cells were reverse transfected with either antisense or precursor miR-148–3p. After 12 hrs of media were repalced with fresh media. Media and cells were collected after 70 hrs of post transfection. Media and cell were processed according to the company kit protocol (abcam, ab108878) to quantify the LP(a). LP(a) concentration obtained in cells lysate and media were normalized with cells total protein and the dataa persented relative to control. One-way annova non paramertic analysis were performed for statical significance analysis. In the figures, significant differences are represented as * P<0.05; **P<0.01; ***P<0.001; ****P<0.0001. Find the attached prism file

### MiRNA expression analysis using LNA array from Exiqon.

The microRNA ready-to-use mouse & rat PCR panels I and II hold 743 different miRNA targets with reference gene assays were used in this study. Total RNA (20 ng) per panel was reverse transcribed using the miRCURY LNA^™^ Universal cDNA synthesis kit (Exiqon). The cDNA was combined with SYBR Green Master Mix, and added to the PCR panels I and II followed by Real-time PCR amplification (Quant Studio 12K Flex Real-Time PCR System). The GenEx version 5 software from MultiD Analyses was used to pre-process and normalize the RT-PCR data. Briefly, mouse & rat panels I and II version 2 layout files were loaded on to the GenEx software. The data was pre-processed according to the software guidelines. Inter-plate calibration was performed using mean values between the panel I and II. The miRNA Cq values larger than 37 were replaced with a blank, all empty rows and undetected miRNAs were removed. Missing ‘NaN’ values were replaced and the data was validated to remove data points for miRNAs, which had less than 60% values from replicates within a group. The final GenEx validated data were further statistically evaluated miRNA fold change were calculated by delta delta CT method.

### Pathway analysis.

Pathway analysis and Gene ontology analysis was performed from huh7 cells RNA-seq data using Ingenuity pathways analysis (IPA) (www.ingenuity.com, Illumina, San Diego, CA).

### Transfection and luciferase assays.

The ON-TARGET *plus* siRNAs against XBP1, ATF4 and ATF6 (SMART pool) were purchased form Dharmacon and were reverse transfected in Huh7cells at a final concentration of 20 nM, using Lipofectamine RNAiMAX (Thermo Fisher Scientific). The miRNA loss-of-function studies were performed using LNA-enhanced antisense miRNA inhibitors (Exiqon) and were forward transfected in huh7 cells for 36 h at a final concentration of 5 nM using Lipofectamine RNAiMAX. Precursor miRNA (Pre-miR) or mimic were purchased from Thermo Fisher Scientific were transfected in Huh7 cells for 36 h at a final concentration of 50 nM using Lipofectamine RNAiMAX. Cells were treated after 32 h of post transfection with Rapamycin (Calbiochem) (1nM) or vehicle (DMSO) for 4 h before protein isolation. Luciferase assays were performed from the whole cell lysates of huh 7 cells transfected with control or miR-148a mimic along with TSC2 3’UTR (wild type or seed mutant) for 36 h. Luciferase activity was measured by Dual-Glo^®^ Luciferase Assay System (Promega Corporation).

### Western blots.

Whole cell lysates were prepared using RIPA buffer (Thermo Fisher Scientific) supplemented with protease inhibitor cocktail (Roche) along with phosphatase inhibitor. Proteins were quantified using the Qubit^™^ Protein Assay Kit (Thermo Fisher Scientific) according to the manufacturer’s protocol. For immunoblotting, 10 μg whole cell lysate per lane was loaded onto a Precast Protein Gels (Bio-Rad) and transferred to nitrocellulose membrane. The membranes were incubated with a rabbit or mouse primary antibody and HRP-conjugated secondary antibody. Amersham ECL Prime reagents were used for the membrane bound antibody detection. The following antibodies were used in this study: anti- mTOR (7C10), anti-phospho-mTOR (Ser2448) (D9C2), anti-4E-BP1 (53H11), anti-phospho-4E-BP1 (Ser65) (D9G10), anti-p70 S6 Kinase (49D7), anti-phospho-p70 S6 Kinase (Thr389), anti-XBP-1s (D2C1F), anti-ATF-4 (D4B8), anti-Raptor (24C12) anti-phospho-Raptor (Ser792), anti-β-Actin (D6A8), Rabbit mAb (Cell Signaling technology), anti-Fatty acid synthase (G-11), anti-ATF6 (F-7), anti-GAPDH, Mouse mAb (Santa Cruz), anti-Tuberin antibody (EP1107Y) Rabbit mAb (abcam).

### Assessment of VLDL production in APOE*3-Leiden.CETP mice.

The following mice procedures were approved by the Central Authority for Scientific Procedures on Animals (CCD) of the Netherlands. Hemizygous APOE*3-Leiden mice were crossbred with homozygous human CETP transgenic mice to generate APOE*3-Leiden.CETP mice. Mice were housed under standard conditions (i.e., group housing, 12 h:12 h light–dark cycle, room temperature of 22 °C) and had *ad libitum* access to food and water. At 8–12 weeks of age, female APOE*3-Leiden.CETP mice were switched from standard chow to a Western-type diet with 25% kcal from cocoa butter and corn oil supplemented with 0.15% cholesterol (ssniff-Spezialdiäten GmbH). After a three-week run-in period, mice that responded well to the diet were divided into four treatment groups (n=8 mice per group), which were balanced for plasma triglyceride and total cholesterol levels. Mice were intraperitoneally injected with vehicle (saline) or 10 mg/kg miR-148a or scrambled LNAs at day 0, and at 5 mg/kg on day 4, 11, 18 and 25. Mice were fasted overnight prior to day 28, and either remained fasted (saline treatment only) or were refed during the first two hours of the light phase. Mice were anesthetized by intraperitoneal administration (10 mL · kg^−1^) of a mixture of Acepromazin (0.63 mg · mL^−1^), Midazolam (0.63 mg · mL^−1^) and Fentanyl (0.03 mg · mL^−1^), followed by subcutaneous administration of 50 μL of the mixture if reflexes re-appeared (approx. every 45 min). Mice were intravenously injected with 10 μCi Tran[35S] (IS-103; Hartmann Analytic) to label newly produced ApoB, and with 5 mL · kg−1 10% Triton WR-1339 (tyloxapol, T0307; Sigma-Aldrich) in PBS 30 min later to block LPL. Just prior to, and 15, 30, 60 and 90 min after injection with Triton WR-1339, blood was collected from the tail vein to measure TG accumulation in plasma. 120 min after injection, mice were exsanguinated via the retroorbital sinus, and the lipoprotein fraction was isolated from serum by aspiration after density gradient ultracentrifugation at d < 1.006 g · mL−1 [25]. 35S activity was determined by liquid scintillation counting (Ultima Gold, PerkinElmer as liquid scintillation cocktail; Tri-Carb 2910 TR, PerkinElmer as scintillation counter) in the lipoprotein fraction, before and after precipitation of ApoB with 2-propanol to calculate VLDL-ApoB production. Triglyceride (10166588130; Roche Diagnostics), cholesterol (11489232216; Roche Diagnostics) and phospholipid (3009; Instruchemie) content was assessed through colorimetric assays.

## Supplementary Material

1

Supplementary Files

This is a list of supplementary files associated with this preprint. Click to download.

- SuppTablesNatComm.xlsx

## Figures and Tables

**Table 1:** Genetic loci associated with the LA / PUFA ratio. Thirteen genetic loci associated with a Bonferroni significant p-gain with the LA/PUFA ratio (log_10_(p-gain) > 11.2); Full summary statistics and further information for these associations are in Table S3. Nearby genes are from the Graham *et al*. annotation; Candidate genes are from Open Targets and ordered by decreasing causal gene score; Likely causal genes were identified based on their direct biochemical link to LA and PUFA metabolism (see annotation in the rightmost column).

SNP	−Log_10_ p-value LA	−Log_10_ p-value PUFA	−Log_10_ p-value LA/PUFA	Log_10_ p-gain LA/PUFA	Nearby genes	Candidate genes	Likely causal gene	Gene annotation; Role of the likely causal in LA and PUFA metabolism
rs174564	169.6	282.5	5,101.7	4,819.2	***FADS2*** *, FADS1*	*FADS1*, ***FADS2****, TMEM258, FEN1, FADS3, BEST1, MYRF, RAB3IL1, FTH1*	***FADS2***	Fatty Acid Desaturase 2; Catalyzes the rate limiting step in the PUFA synthesis from essential linoleic acid (LA) (18:2n-6) and alpha-linolenic acid (ALA) (18:3n-3) precursors^62^
rs12928099	3.1	20.1	170.4	150.3	*RRN3, PDXDC1, NTAN1*	*NTAN1, RRN3, NPIPA5, NPIPA1, PDXDC1, MYH11*	***PLA2G10***	Phospholipase A2 Group X; Hydrolyzes extracellular phospholipids at the sn-2 position, prefers PUFA chains over saturated fatty acyls^63^
rs673335	3.7	36.0	67.9	32.0	*RN7SL786P, MOGAT2-RN7SL786P*	*DGAT2, UVRAG*	***MOGAT2*** ^+^	Monoacylglycerol O-Acyltransferase 2; Resynthesis of TAGs from dietary fat, has a preference toward MAGs containing unsaturated fatty acids in an order of C18:3 > C18:2 > C18:1 > C18:0 at the sn-2 position^64^
rs1123571	5.0	0.0	29.8	24.8	*MORN1, RER1*	*RER1, PEX10*, ***PLCH2***	***PLCH2***	Phospholipase C Eta 2; Hydrolyses phosphatidylinositol 4,5-bis-phosphate to generate two second messengers, inositol 1,4,5-trisphosphate and diacylglycerol^65^
rs7924036	5.6	0.2	26.0	20.5	*PRELID1P3, JMJD1C*	*REEP3, NRBF2*	-	No causal gene identified
rs2003892	0.4	4.0	23.9	19.9	***CPT1A***	*MRPL21, IGHMBP2, TPCN2, MRGPRF*, ***CPT1A****, MRGPRD*	***CPT1A***	Carnitine Palmitoyltransferase 1A; Catalyzes the transfer of the acyl group of long-chain fatty acid-CoA conjugates onto carnitine for mitochondrial uptake and subsequent beta-oxidation in the mitochondrion^66^
rs72997616	5.1	36.0	55.0	19.0	***DGAT2*** *, CTD-2530H12.1*	***DGAT2*** *, UVRAG*	***DGAT2***	Diacylglycerol O-Acyltransferase 2; Catalyzes the terminal and only committed step in triacylglycerol synthesis by using diacylglycerol and fatty acyl CoA as substrates^67^
rs10779835	1.7	0.7	16.5	14.9	***GALNT2***	***GALNT2***	***GALNT2***	Polypeptide N-Acetylgalactosaminyl-transferase 2; Alters HDL metabolism through O-linked glycosylation of APOC-III, ANGPTL3 and PLTP^68^
rs267738	0.3	3.0	17.9	14.8	*RP11–316M1.3, SETDB1, RP11–316M1.12*, ***CERS2***	*CTSS*, ***CERS2****, GOLPH3L, CTSK, CDC42SE1, ADAMTSL4, HORMAD1, ARNT, ANXA9*	***CERS2***	Ceramide Synthase 2; Catalyzes the transfer of the acyl chain from acyl-CoA to a sphingoid base, with high selectivity toward very-long-chain fatty acyl-CoA (chain length C22-C27)^69^
rs267733	1.1	0.9	14.7	13.6	*ANXA9*	*ANXA9, CTSK, CDC42SE1, GOLPH3L, GABPB2, HORMAD1, ADAMTSL4*	-	No causal gene identified
rs10896373	0.2	3.2	16.3	13.1	*CPT1A-RP11–757G1.6*, ***CPT1A***	*IGHMBP2, MRPL21, CPT1A, TESMIN, TPCN2, MRGPRF*	***CPT1A***	This is a second independent genetic signal at the CPT1A locus, see above.
rs3747207	3.9	0.3	16.2	12.2	***PNPLA3***	*SAMM50*, ***PNPLA3***	***PNPLA3***	Patatin Like Phospholipase Domain Containing 3; Catalyzes acylation of 2-lysophosphatidic acid to phosphatidic acid, acyl donors are long chain (at least C16) fatty acyl-CoAs, including linoleoyl-CoA^70^
rs9296406	2.0	0.2	13.4	11.4	*PEX6*	*GNMT, PEX6, CNPY3, PPP2R5D, MRPL2, RPL7L1, BICRAL*	-	No causal gene identified

+ MOGAT2 and DGAT2 are encoded in proximity, the likely causal gene for these two identifications is based on genetic linkage using snipa.org

## Data Availability

All analyzed UK Biobank data was obtained through the UKB RAP system under application reference number 43418 and 588633. All generated data are provided with the paper or are available online on FigShare (https://doi.org/10.6084/m9.figshare.19728991.

## Code availability statement

Only publicly available software was used in data analysis (R and Rstudio).
