# Supplementary material for: Ratio-Driven Lipoprotein Mapping Refines Genetic Pathways of Cardiometabolic Risk
Source: Res Sq. 2026 Jan 7:rs.3.rs-8475327. Preprint. [Version 1] doi: 10.21203/rs.3.rs-8475327/v1 (PMC12803353; doi:10.21203/rs.3.rs-8475327/v1)
Supplement: 1 [file NIHPPRS8475327V1-supplement-1.pdf]

SUPPLEMENTARY TABLES

	A	B
1	Supplementary Tables for **"Suhre et al., Ratio-driven lipoprotein fine-mapping of genetic lipid risk loci uncovers**	
2		
3	**Table**	**Content**
4	ST1	List and annotation of the 168 NMR traits
5	ST2	List of 81 ratios proposed by Nightingale (NightRatios)
6	ST3	Lead association data for all computed models (based on 2,624 entries of ST3 by Graham et al.), incl. replication in non-Caucasians
7	ST4	Scaled -log10(p-value) for 446 genetic loci and 168 traits
8	ST5	Scaled effect sizes (beta) for 446 genetic loci and 168 traits
9	ST6	208 ratios x 347 variants (unique ratios that were strongest association in at least one association)
10	ST7	Summary statistics for all 208 ratios x 347 variants associations with log10(p-gain) > 11.2
11	ST8	71 ratios x 84 LPmtr loci
12	ST9	18 key ratios x 84 LPmtr loci
13	ST10	Associations of ratios related to Total.Esterified.Cholesterol / Total.Cholesterol for 347 loci
14	ST11	Associations of ratios related to Concentration.of.HDL.Particles / Total.Concentration.of.Lipoprotein.Particles for 347 loci
15	ST12	Association of lipid particle composition with LPmtr genes
16	ST13	Overlapping UKB-PPP pQTLs (from Sun et al., Nature, 2023)
17	ST14	Count of overlapping associations with LPmtr genes that are on the OLINK panel
18	ST15	Selected summary statistics for NMR trait associations discussed in the paper

SUPPLEMENTARY DATA

The complete summary statistics for all NMR-trait and NMR-ratio associations are available on FigShare https://doi.org/10.6084/m9.figshare.19728991.

SUPPLEMENTARY FIGURES

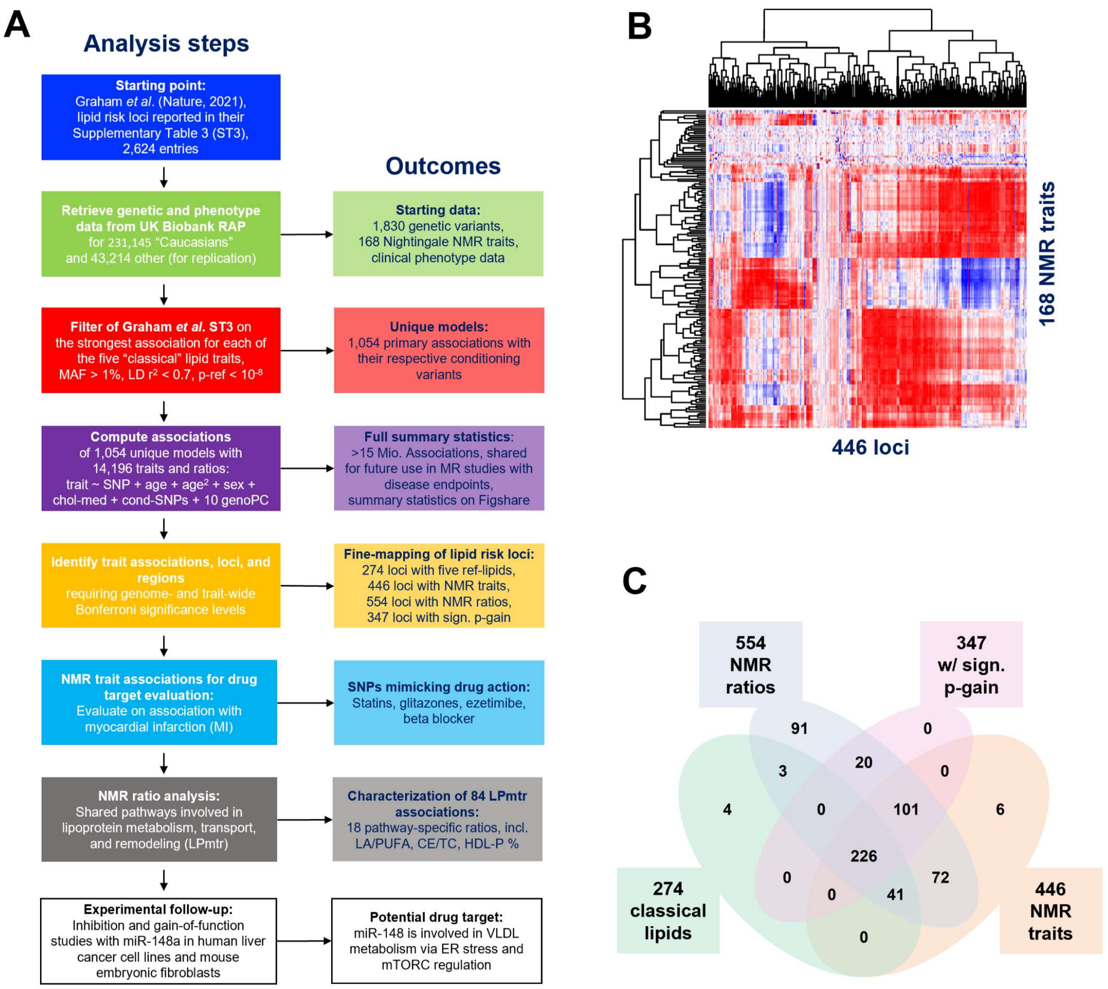

Figure S1. Study design and outcomes. (**A**) Flow chart of the present study; (**B**) Heatmap of the effect estimates (beta), scaled by division using the largest absolute beta value and aligning the directionality (sign of beta) such that the strongest beta at each locus has a positive association with the respective effect allele of that variant; Blue colors represent negative associations and red positive associations; (**C**) Heatmap of log10(p-gain) for the association of 208 NMR ratios at the 374 genetic loci, scaled by division using the largest log10(p-gain) at each locus; Darker colors represent stronger associations; (**D**) Venn diagram of loci reaching Bonferroni significance; A total of 564 out of the 1,054 lead variants from Graham et al. associated at the respective Bonferroni significance level with at least one of the five lipids (274 variants), one of the NMR traits (446 variants), or the NMR ratios (554 variants); The p-gain with ratios was significant at a Bonferroni level for 347 variants.

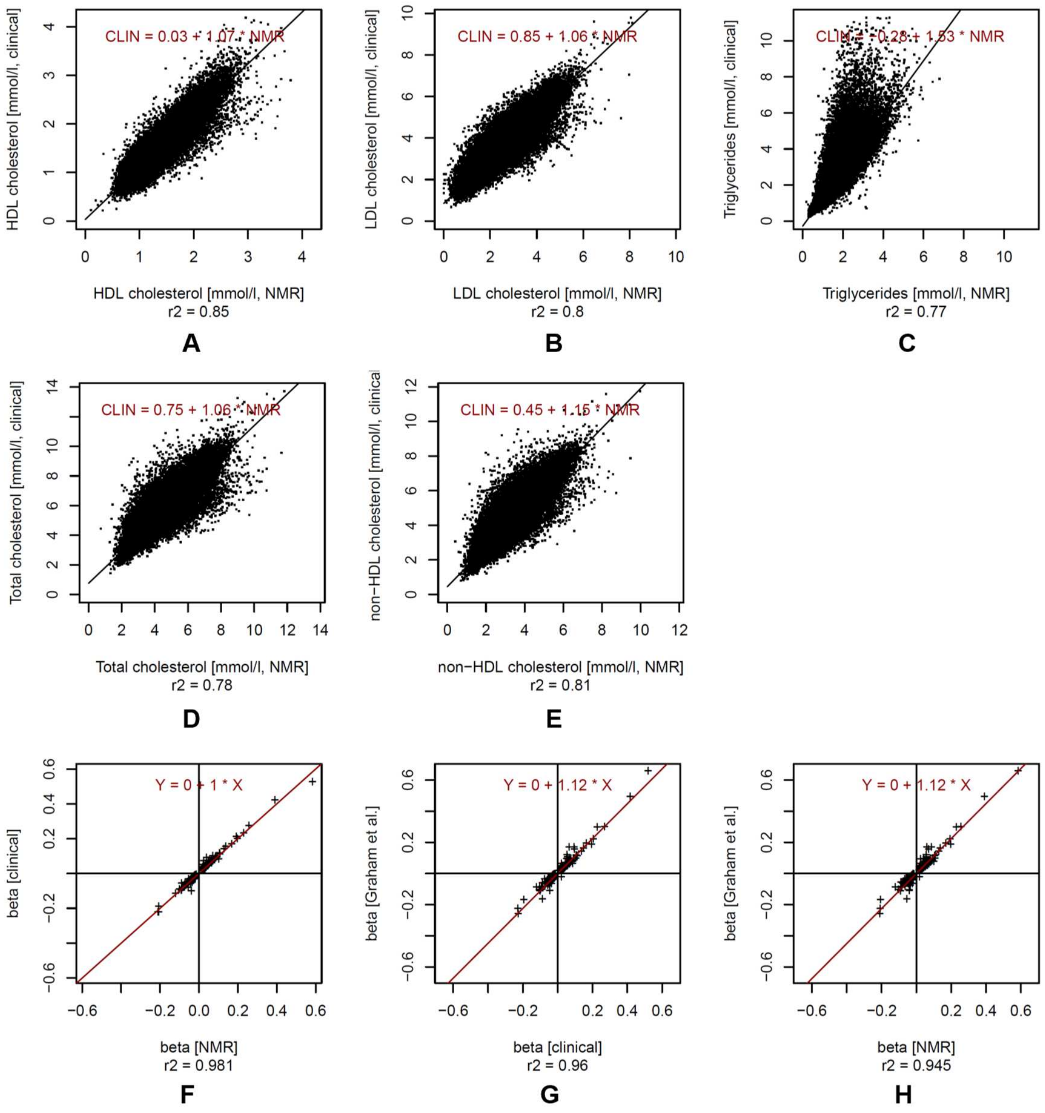

Figure S2: Comparison of lipid measures obtained using the NMR platform and clinical biochemistry. Scatterplots of the five lipid traits measured by NMR and using clinical biochemistry (A-E); Scatterplots of the effect sizes (beta) for the associations of the 274 lead variants with the lead lipid trait reported in ST3 by Graham *et al*. obtained in different ways: using the five lipid traits from the Nightingale NMR data (beta [NMR]) compared to using the clinical biochemistry data (beta [clinical]) on the 231,000 UKB samples (F); using the clinical biochemistry data compared to estimates from ST3 in Graham *et al*. (beta [Graham *et al*.]) (G); using the NMR data compared to Graham *et al.* (H).

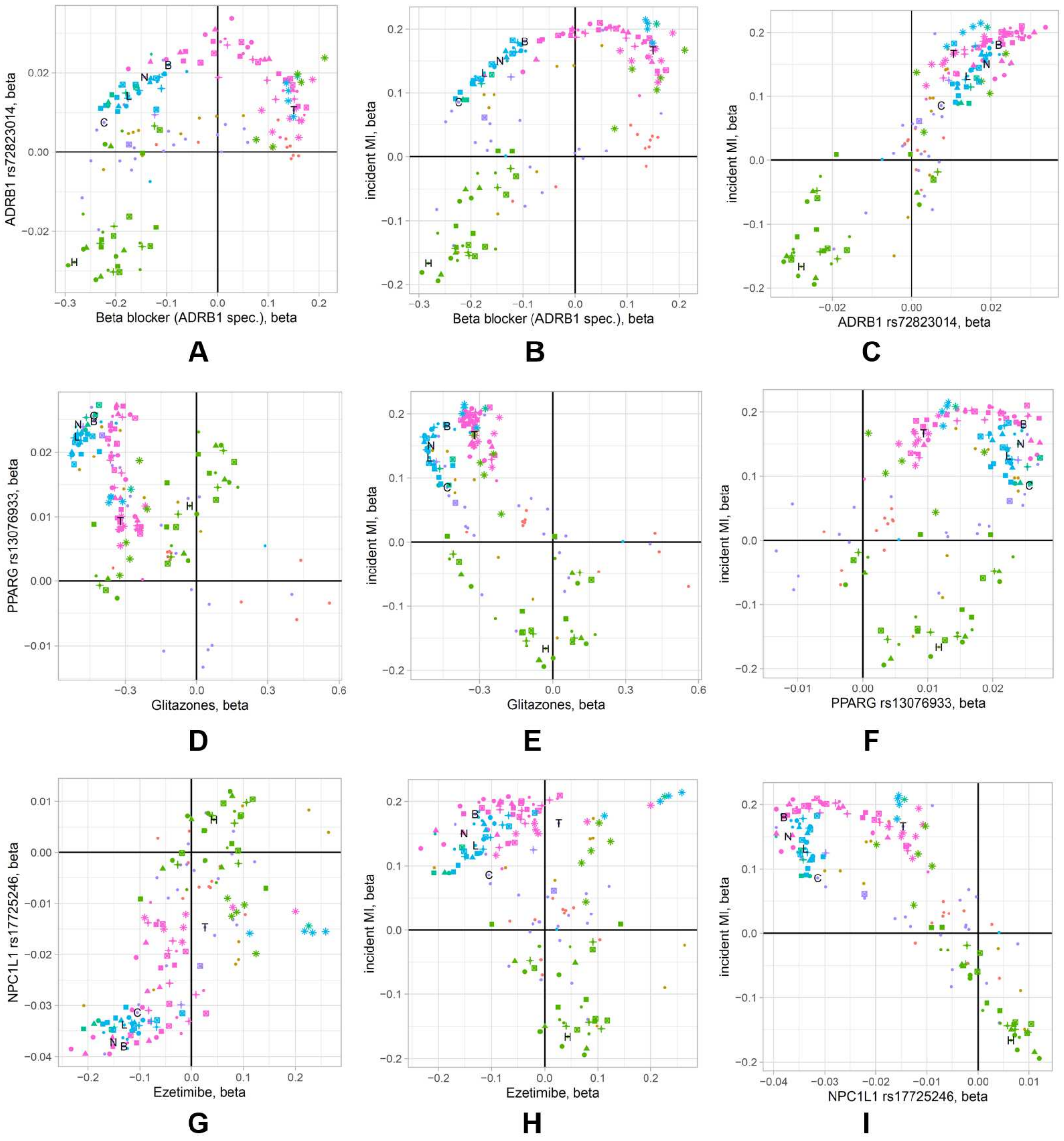

Figure S3: Genetic variants mimicking drug effects and their corresponding drug usage with NMR traits. Scatterplots of the effect estimates for the associations of three variants near genes coding for established drug targets (ADRB1, NPC1L1, PPARG) with the 168 NMR traits and the effect sizes for the associations of the corresponding drugs (beta blocker, ezetimibe, glitazones) with the 168 NMR traits; The effect estimates are based on 17,166 users of ADRB1 specific beta blockers, 1,427 users of ezetimibe, and 951 users of glitazones, out of which 478 used pioglitazone and 473 used rosiglitazone.

A

```
Call:
lm(formula = LDLR ~ CE)

Residuals:
     Min       1Q   Median       3Q      Max 
-2.42527 -0.41377 -0.01435  0.39279  3.02031 

Coefficients:
            Estimate Std. Error t value Pr(>|t|)    
(Intercept) -0.51156    0.02220  -23.04   <2e-16 ***
CE           0.30985    0.01274   24.32   <2e-16 ***
---
Signif. codes:  0 ‘***’ 0.001 ‘**’ 0.01 ‘*’ 0.05 ‘.’ 0.1 ‘ ’ 1

Residual standard error: 0.6219 on 24774 degrees of freedom
  (524 observations deleted due to missingness)
Multiple R-squared:  0.02331,	Adjusted R-squared:  0.02327 
F-statistic: 591.3 on 1 and 24774 DF,  p-value: < 2.2e-16
```

B

```
Call:
lm(formula = LDLR ~ TC)

Residuals:
     Min       1Q   Median       3Q      Max 
-2.46241 -0.40890 -0.01458  0.38790  3.06479 

Coefficients:
            Estimate Std. Error t value Pr(>|t|)    
(Intercept) -0.80934    0.02763  -29.29   <2e-16 ***
TC           0.38112    0.01258   30.31   <2e-16 ***
---
Signif. codes:  0 ‘***’ 0.001 ‘**’ 0.01 ‘*’ 0.05 ‘.’ 0.1 ‘ ’ 1

Residual standard error: 0.6179 on 24774 degrees of freedom
  (524 observations deleted due to missingness)
Multiple R-squared:  0.03575,	Adjusted R-squared:  0.03571 
F-statistic: 918.5 on 1 and 24774 DF,  p-value: < 2.2e-16
```

C

```
Call:
lm(formula = LDLR ~ CE + TC)

Residuals:
     Min       1Q   Median       3Q      Max 
-2.19569 -0.33686 -0.01602  0.31302  2.93121 

Coefficients:
             Estimate Std. Error t value Pr(>|t|)    
(Intercept)  -8.62578    0.07497  -115.1   <2e-16 ***
CE          -17.70107    0.16182  -109.4   <2e-16 ***
TC           17.92699    0.16073   111.5   <2e-16 ***
---
Signif. codes:  0 ‘***’ 0.001 ‘**’ 0.01 ‘*’ 0.05 ‘.’ 0.1 ‘ ’ 1

Residual standard error: 0.5074 on 24773 degrees of freedom
  (524 observations deleted due to missingness)
Multiple R-squared:  0.3498,	Adjusted R-squared:  0.3497 
F-statistic:  6664 on 2 and 24773 DF,  p-value: < 2.2e-16
```

D

```
Call:
lm(formula = LDLR ~ CE.by.TC)

Residuals:
     Min       1Q   Median       3Q      Max 
-2.33689 -0.33893 -0.01356  0.31972  2.98098 

Coefficients:
             Estimate Std. Error t value Pr(>|t|)    
(Intercept)  -8.35428    0.07462  -112.0   <2e-16 ***
CE.by.TC    -18.17849    0.16182  -112.3   <2e-16 ***
---
Signif. codes:  0 ‘***’ 0.001 ‘**’ 0.01 ‘*’ 0.05 ‘.’ 0.1 ‘ ’ 1

Residual standard error: 0.5122 on 24774 degrees of freedom
  (524 observations deleted due to missingness)
Multiple R-squared:  0.3375,	Adjusted R-squared:  0.3374 
F-statistic: 1.262e+04 on 1 and 24774 DF,  p-value: < 2.2e-16
```

Figure S4: Output of R linear model analyses of the association of LDLR with cholesterol. R-output for linear models of LDLR levels (Olink) against log-scaled concentrations of cholesteryl ester (CE) (A), total cholesterol (TC) (B), a linear combination of both (C), and the difference of the log-scaled variables (CE.by.TC), which is equivalent to the log-scaled ratio CE / TC (D); The readouts reported by Olink are relative units and are proportional to the logscale of the blood circulating LDLR levels; LDLR is presumably present in the form of cleaved/soluble epitopes (sLDLR); Note the opposite effect estimates that are of equal magnitude for CE and TC in model (C) and that match the magnitude of the effect estimate for CE.by.TC in model (D), suggesting that in this case using ratios is equivalent to considering one trait playing the role of a covariate for shared non-genetic variance, as we discuss in ref [18]; Here, large variance in TC arguably masks the association of CE with LDLR, which can be revealed when considering the ratio of esterified cholesterol by total cholesterol; See **Figure 3H-I** for scatterplots between CE and TC and between CE / TC and LDLR.

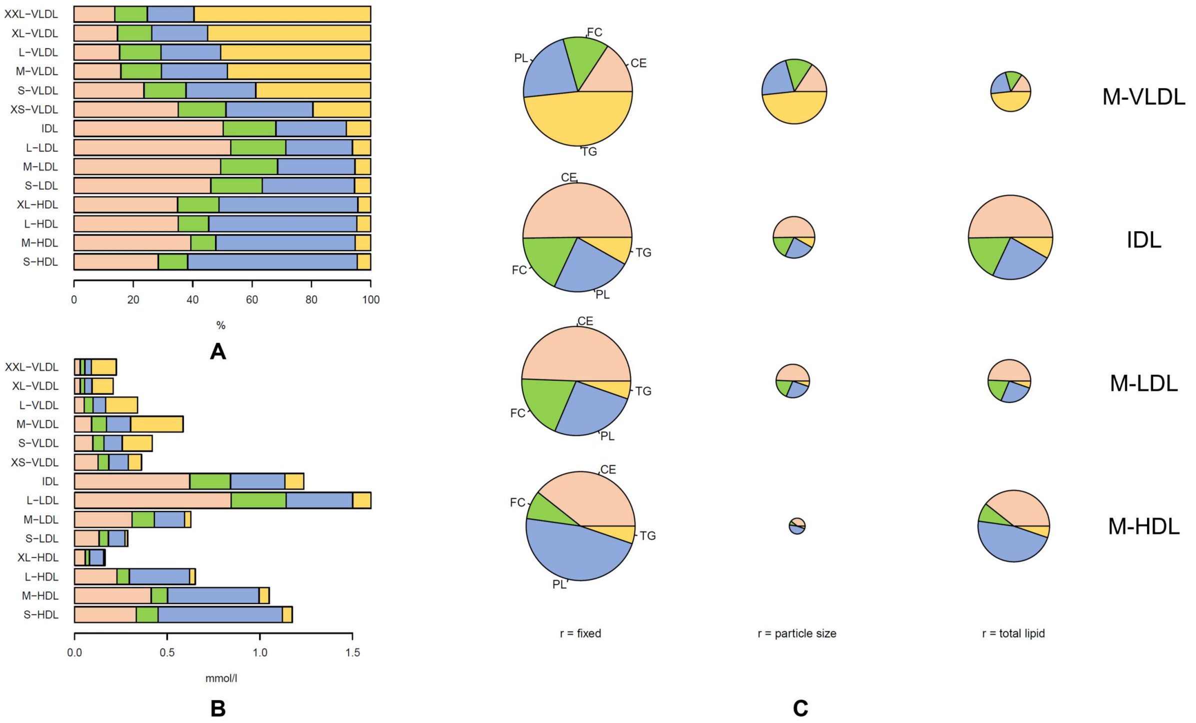

Figure S5: Visual representations of the lipid composition of the 14 lipoprotein size classes. VLDL particles are rich in triglycerides, while IDL and LDL particles are rich in cholesterol, and HDL particles have the highest phospholipid content (A); Most lipids are present in the form of IDL and large LDL particles and also as small and medium sized HDL particles (B); Selected lipoprotein classes scaled by particle size and total lipid content (C); Note that Nightingale reports six lipid traits per size class, while only four are shown here; Two further variables are numerically calculated, that is, total cholesterol as the sum of free and esterified cholesterol and the total lipid content as the sum of total cholesterol, triglycerides and phospholipids.

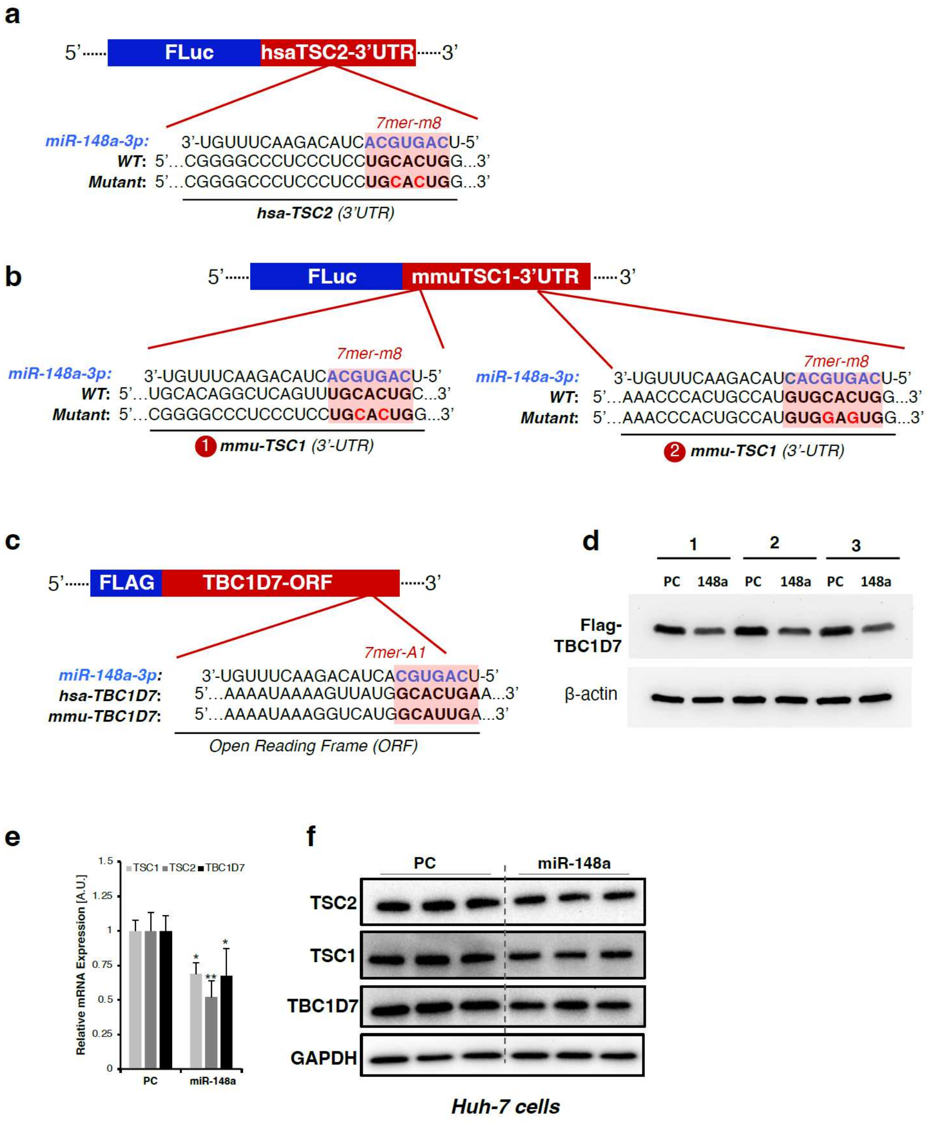

Figure S7: MicroRNA-148a directly inhibits components of the tuberous sclerosis complex (TSC) in human and mice. (a)TSC2 3'-UTR sequence with wildtype and mutant seed regions highlighted. Luciferase reporter activity assay of constructs harboring intact TSC2 3′ UTR or mutated seed regions for miR-148a, after transfection with miR-148a mimic. A scrambled miRNA mimic was used as control. (c)…(d) Luciferase reporter activity assay of constructs harboring intact mouse TSC1 3′ UTR or mutated seed regions for miR-148a, after transfection with miR-148a mimic. A scrambled miRNA mimic was used as control. (e) Expression levels of TSC1, TSC2 and TBC1D7 in huh 7 cells transfected with CTL mimic and miR-148a mimic for 36 h. (B)Immunoblot analysis of TSC2 in huh 7 cells transfected with CTL mimic and miR-148a mimic for 36 h.(f)Immunoblot analysis of TSC1/TSC2/TBC1D7 in mice treated with miR-148a. Error bars are mean ±SD from three independent experiments. *P<0.05 **P<0.01.

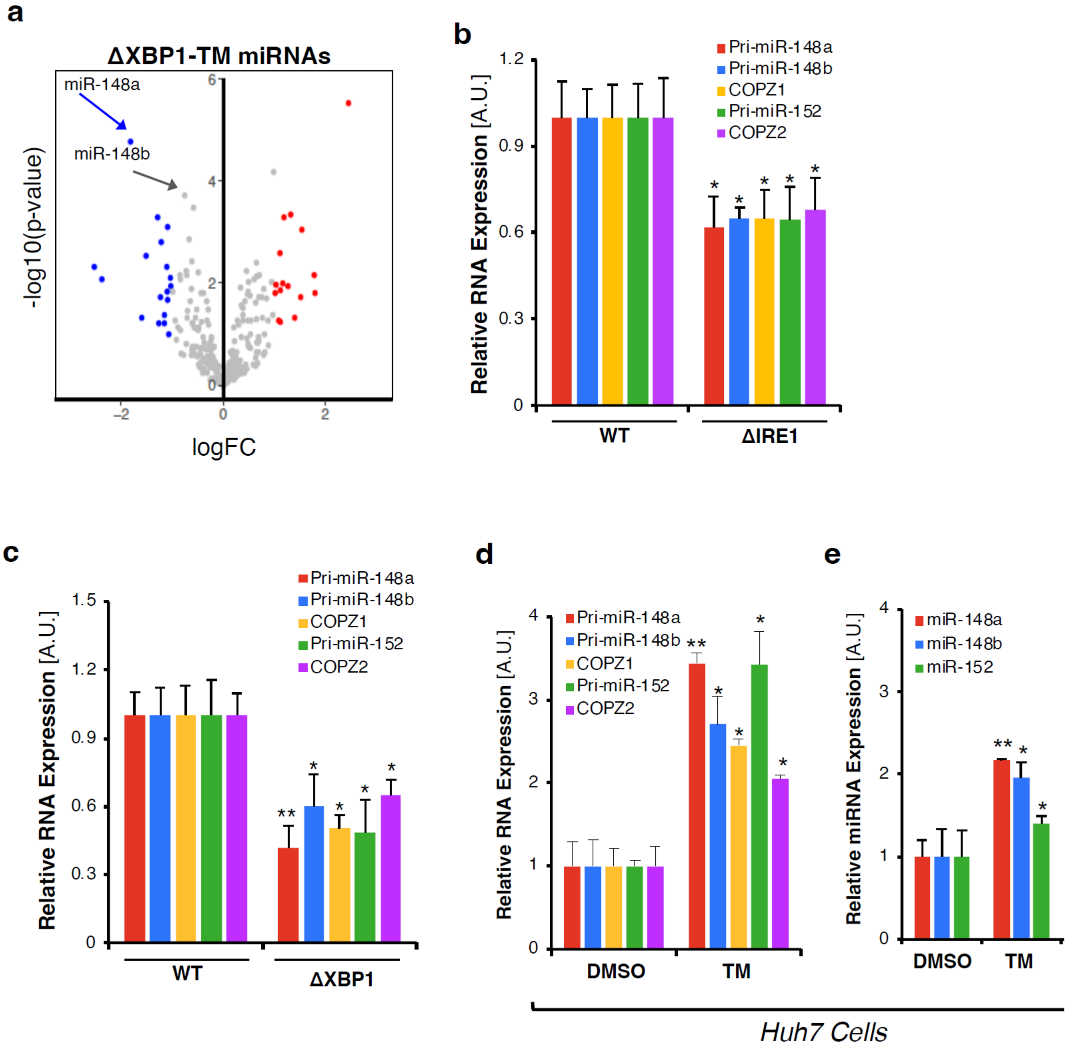

Figure S8: IRE1-XBP1 axis dependent regulation of miR-148 microRNAs and their COPZ host genes in mice and human liver cells. (a-d) Quantitative RT-PCR analysis of primary transcript of microRNA-148 isoforms in IRE1α, XBP1 WT and liver-specific deficient mice (n=6). (F-G) Relative expression of primary and mature miRNA-148 isoforms levels in huh7 human hepatocytes treated with tunicamycin measured by qRT-PCR. Error bars are SEM n=3. *P<0.05; **P<0.01; ***P<0.005.

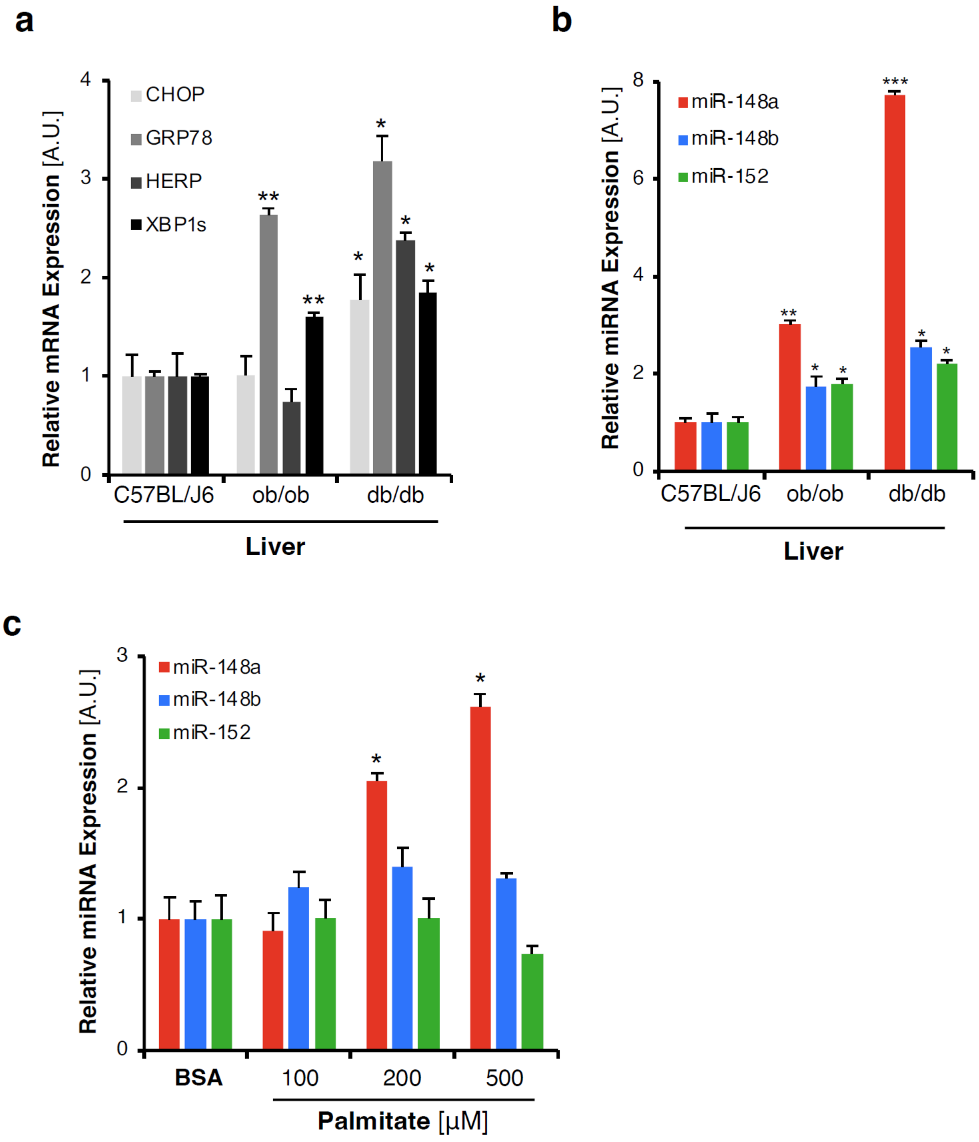

Figure S9: Increased microRNA 148 expression in major obese mouse models and in response to palmitate in human liver cells indicate its metabolic relevance during ER-stress. (A)XBP1s and ER stress markers measured by qRT-PCR in lep(ob/ob), lepr (db/db) compared with C57BL/6J wild-type mice. (B) Analysis of mature microRNA in lean and genetic obese mouse models measured by qRT-qPCR normalized to miR-423. Errors bars are SEM n=6 *P<0.05; **P<0.01.

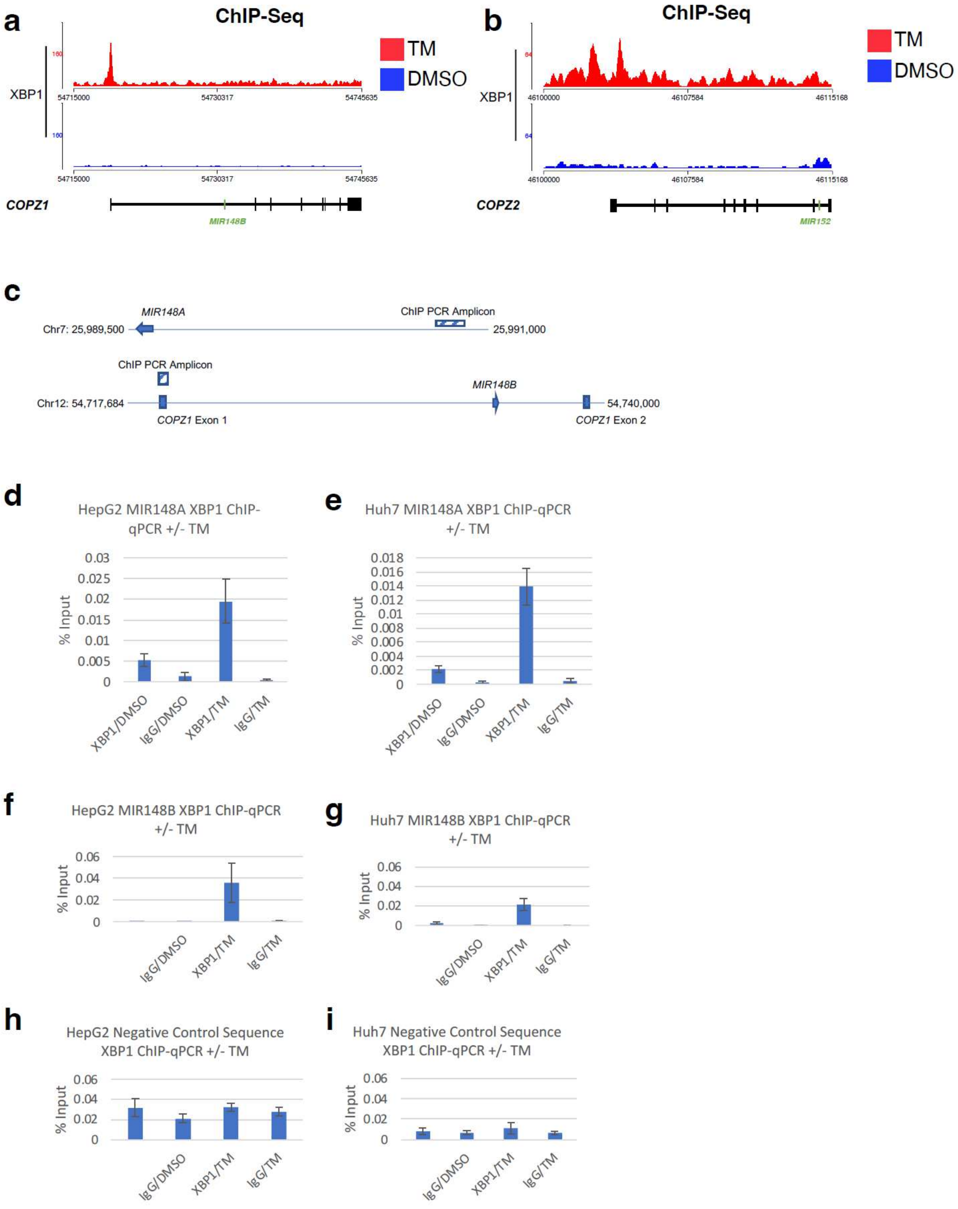

Figure S10. Enriched XBP-1 binding to COPZ2 and COPZ1 gene regions in response to ER-Stress. (a-b) Density profiles generated from ChIP-Seq data showing XBP1 binding to genomic loci harboring microRNA 148 isoforms in ER-stress induced breast cancer cells. (c) Schematic depiction of the locus targeted for ChIP analysis. (**d-e**) ChIP analysis showing enrichment for XBP-1 binding to miR148a locus in response to TM in HepG2 (d) and Huh7 (e) cells. (**f-g**) ChIP analysis showing enrichment for XBP-1 binding to COPZ1/miR148b locus in response to TM in HepG2 (f) and Huh7 (g) cells, as compared to IgG control. The same patterns of XBP1 enrichment were not observed at a negative control sequence (**h-i**).

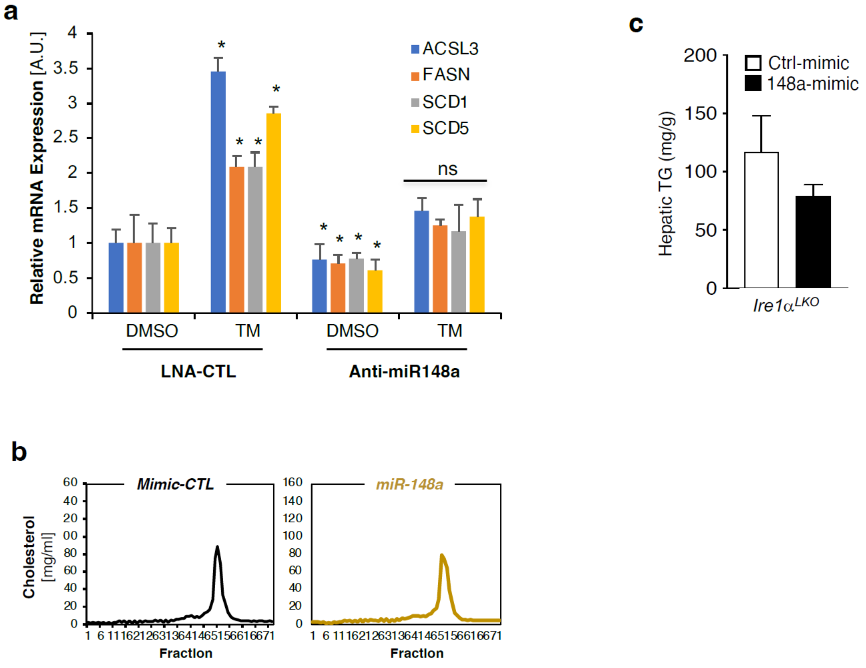

Figure S11. miR-148a depletion in the absence and presence of ER-stress modulates hepatic SREBP1c target genes. (a) Relative expression of SREBP1 target mRNAs in huh7 cells transfected with CTL LNA or miR-148a LNA in presence of absence of TM induced ER stress. (b) Changes in hepatic TG content in miR148a treated IRE1a deficient mice. (c) FPLC analysis of plasma cholesterol levels from miR-148 treated IRE1a deficient mice.

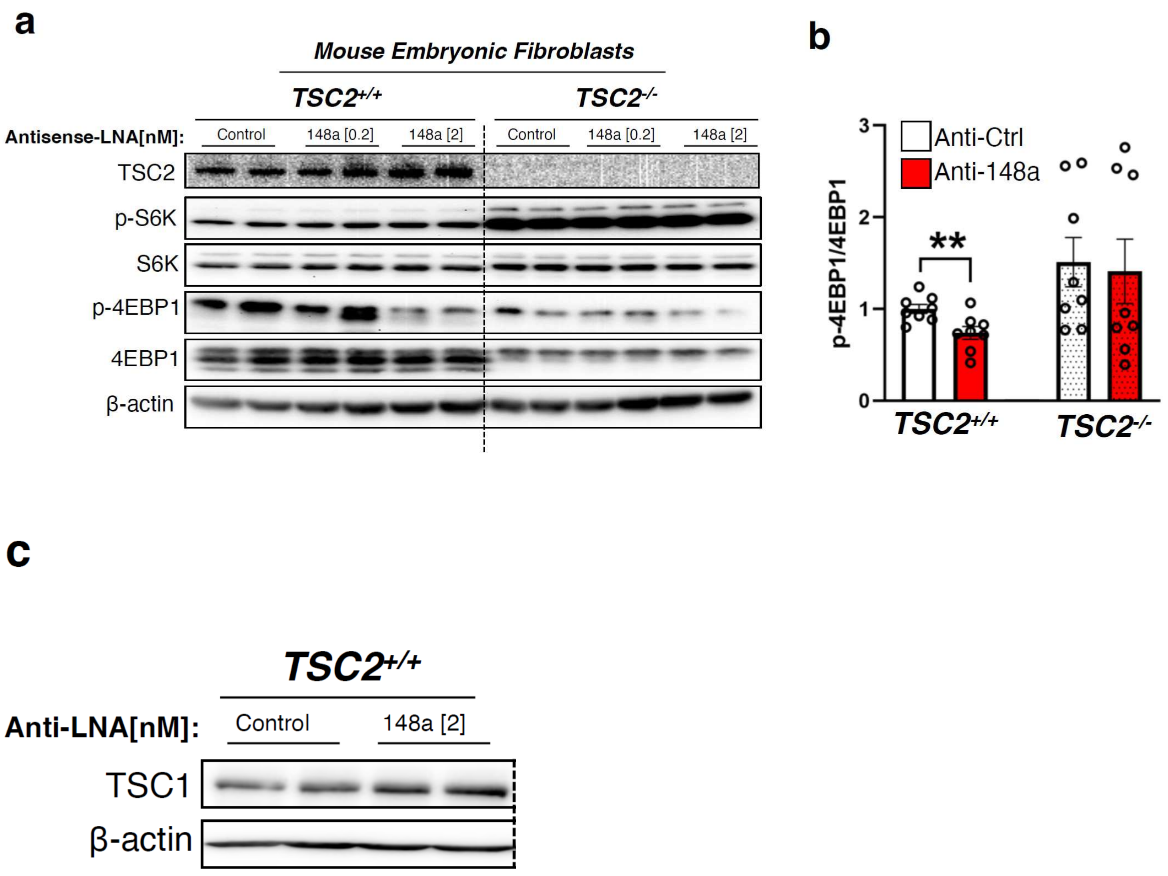

Figure S12: miR-148a depletion effect on MTORC1 activity in naïve and TSC2 deficient mouse embryonic fibroblasts (MEFs). (a-b) Analysis of MTORC1 target protein 4EBP1 in response to LNA Anti-miR148a mediated inhibition of miR-148a. (c) Analysis of miR-148a target TSC1 in respsone to miR-148a inhibition in both naïve and TSC2 deficient cells.
